# Alkaline ceramidase 2 and its bioactive product sphingosine are novel regulators of the DNA damage response

**DOI:** 10.18632/oncotarget.7825

**Published:** 2016-03-01

**Authors:** Ruijuan Xu, Kai Wang, Izolda Mileva, Yusuf A. Hannun, Lina M. Obeid, Cungui Mao

**Affiliations:** ^1^ Department of Medicine, State University of New York at Stony Brook, Stony Brook, NY 11794, USA; ^2^ Stony Brook Cancer Center, State University of New York at Stony Brook, Stony Brook, NY 11794, USA; ^3^ Lipidomics Core Facility, State University of New York at Stony Brook, Stony Brook, NY 11794, USA; ^4^ Ralph H. Johnson Veterans Administration Hospital, Stony Brook, NY 11794, USA

**Keywords:** ceramide, Golgi, p53, programmed cell death, reactive oxygen species

## Abstract

Human cells respond to DNA damage by elevating sphingosine, a bioactive sphingolipid that induces programmed cell death (PCD) in response to various forms of stress, but its regulation and role in the DNA damage response remain obscure. Herein we demonstrate that DNA damage increases sphingosine levels in tumor cells by upregulating alkaline ceramidase 2 (ACER2) and that the upregulation of the ACER2/sphingosine pathway induces PCD in response to DNA damage by increasing the production of reactive oxygen species (ROS). Treatment with the DNA damaging agent doxorubicin increased both ACER2 expression and sphingosine levels in HCT116 cells in a dose-dependent manner. ACER2 overexpression increased sphingosine in HeLa cells whereas knocking down ACER2 inhibited the doxorubicin-induced increase in sphingosine in HCT116 cells, suggesting that DNA damage elevates sphingosine by upregulating ACER2. Knocking down ACER2 inhibited an increase in the apoptotic and necrotic cell population and the cleavage of poly ADP ribose polymerase (PARP) in HCT116 cells in response to doxorubicin as well as doxorubicin-induced release of lactate dehydrogenase (LDH) from these cells. Similar to treatment with doxorubicin, ACER2 overexpression induced an increase in the apoptotic and necrotic cell population and PARP cleavage in HeLa cells and LDH release from cells, suggesting that ACER2 upregulation mediates PCD in response to DNA damage through sphingosine. Mechanistic studies demonstrated that the upregulation of the ACER2/sphingosine pathway induces PCD by increasing ROS levels. Taken together, these results suggest that the ACER2/sphingosine pathway mediates PCD in response to DNA damage through ROS production.

## INTRODUCTION

Mammalian cells respond to DNA damage by undergoing cell cycle arrest in order to repair damaged DNA or by initiating programmed cell death (PCD) if the damaged DNA is irreparable [1]. These coordinated biological processes, known as the DNA damage response (DDR), ensure not to pass on the damaged genome from mother cells to daughter cells, thereby playing a protective role in the genomic integrity [2]. Because dysregulation of the DDR results in genomic instability and contributes greatly to both cancer development and progression [3], understanding the molecular mechanism of this biological process is of great importance.

Numerous studies demonstrated that mammalian cells respond to DNA damage by increasing the levels of bioactive sphingolipids, including ceramides [4–14], sphingosine (SPH) [15], and sphingosine-1-phosphate (S1P) [16], which have been implicated in various cellular responses. Both ceramides [4–12] and SPH [15] have been shown to induce cell growth arrest and/or PCD in response to various forms of stress, including DNA damage. In contrast to ceramides and SPH, S1P has been shown to mainly promote cell proliferation and survival [17–19] although it may also exert the opposing effects on certain cell types [20, 21]. The role and regulation of ceramides and S1P in the DDR have been intensively studied, whereas much remains unclear about the regulation and role of SPH in the DDR.

In mammalian cells, ceramides are generated via multiple pathways catalyzed by various enzymes, including sphingomyelinases (SMPDs) [22] and (dihydro) ceramide synthases (CERSs) [23] (Supplementary Figure S1). SPH is essentially generated from the hydrolysis of ceramides by the action of ceramidases [24] (Supplementary Figure S1). To date, five human ceramidase genes have been identified, including *ASAH1*, *ASAH2*, *ACER1*, *ACER2*, and *ACER3*, and their protein products are classified as the acid (ASAH1), neutral (ASAH2), and alkaline ceramidase (ACER1–3) subtypes according to their pH optima for their catalytic activity [24]. SPH is in turn phosphorylated by SPHK1 and SPHK2 to form S1P [25] (Supplementary Figure S1). Increasing studies have demonstrated that ceramidases may play an important role in regulating the DDR by controlling the levels of bioactive sphingolipids. Morales *et al.* [26] demonstrated that treatment with daunorubicin, a DNA damaging chemotherapeutic agent, transiently increases acid ceramidase activity in liver cancer cells and that this activity increase attenuates daurorubicin-induced programmed cell death probably by inversely regulating cellular levels of ceramide and S1P. Cheng et al. [27] demonstrated that the acid ceramidase ASAH1 is upregulated by ionizing radiation (IR), a potent DNA damaging insult, in tumor cells and that its upregulation protects tumor cells from IR-induced apoptosis by reducing ceramides and/or increasing S1P. Wu *et al.* [28] showed that the mouse neutral ceramidase Asah2 was downregulated in transformed murine endothelial cells by Gemcitabine, a DNA damaging chemotherapeutic agent, and that its downregulation mediates cell cycle arrest probably by increasing the cellular levels of ceramides. Uchida *et al.* [29] found that ultraviolet radiation downregulates both ASAH1 and ASAH2 in human epidermal keratinocytes and that the downregulation of these ceramidases mediates apoptosis probably by elevating ceramides and/or reducing S1P. These results suggest that ASAH1 and ASAH2 play an important role in the DDR by regulating ceramides and/or S1P other than SPH. Intriguingly, although SPH has been long known to mediate PCD in cells in response to DNA damage [15], the ceramidase (s) responsible for SPH generation in response to DNA damage has (have) not been identified.

In this study, with a qPCR array that simultaneously quantifies mRNA levels of major enzymes involved in the metabolism of sphingolipids, we identify ACER2, a Golgi alkaline ceramidase [30], as the major sphingolipid-metabolizing enzyme whose expression is markedly upregulated by DNA damage. We provide ample evidence that ACER2 is the ceramidase responsible for the SPH rise in response to DNA damage. More importantly, we demonstrate that the upregulation of the ACER2/SPH pathway mediates PCD in response to DNA damage by inducing the production of reactive oxygen species (ROS), thus, offering novel insights into the molecular mechanism of the DDR.

## RESULTS

### The DNA damaging agent doxorubicin (DXR) increases the levels of SPH and S1P in human tumor cells

**Figure 1:**
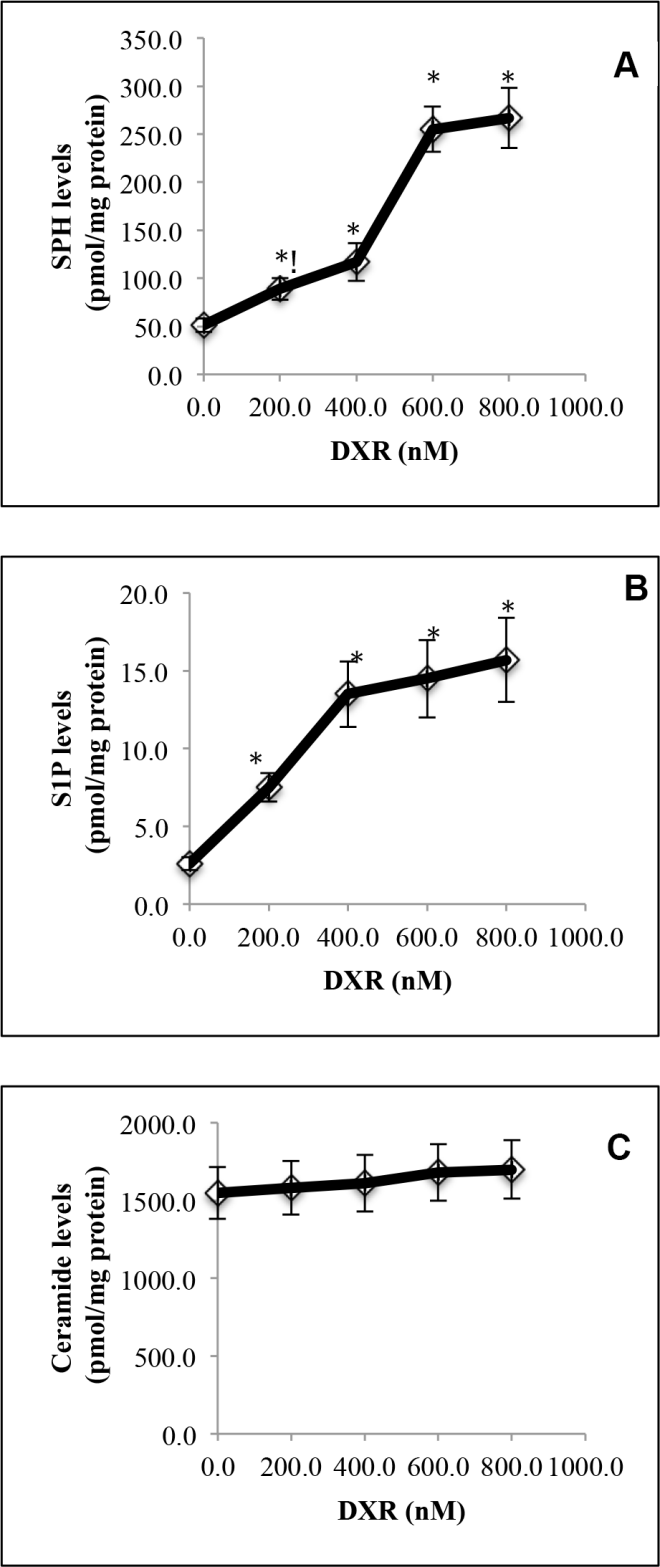
DNA damage by doxorubicin increases SPH and S1P levels in HCT116 cells HCT116 cells were treated with DXR at 200, 400, 600 or 800 nM or DMSO for 24 h before the levels of SPH (**A**), S1P (**B**), and ceramides (**C**) were determined by LC-MS/MS. Data represent mean values ± SD of 3 independent experiments. *p-values of DXR at different concentrations versus (vs) DMSO.

With LC-MS/MS, we demonstrated that treatment with the DNA damaging agent doxorubicin (DXR) increased the levels of SPH (Figure 1A) and S1P (Figure 1B) in HCT116 cells in a dose-dependent manner. Unexpectedly, treatment with DXR only slightly increased the levels of ceramides in HCT116 cells (Figure 1C). These results suggest that cells respond to the DNA damaging agent DXR by increasing the levels of both SPH and S1P and to a lesser extent, ceramides in HCT116 cells.

### DNA damage upregulates ACER2

**Figure 2:**
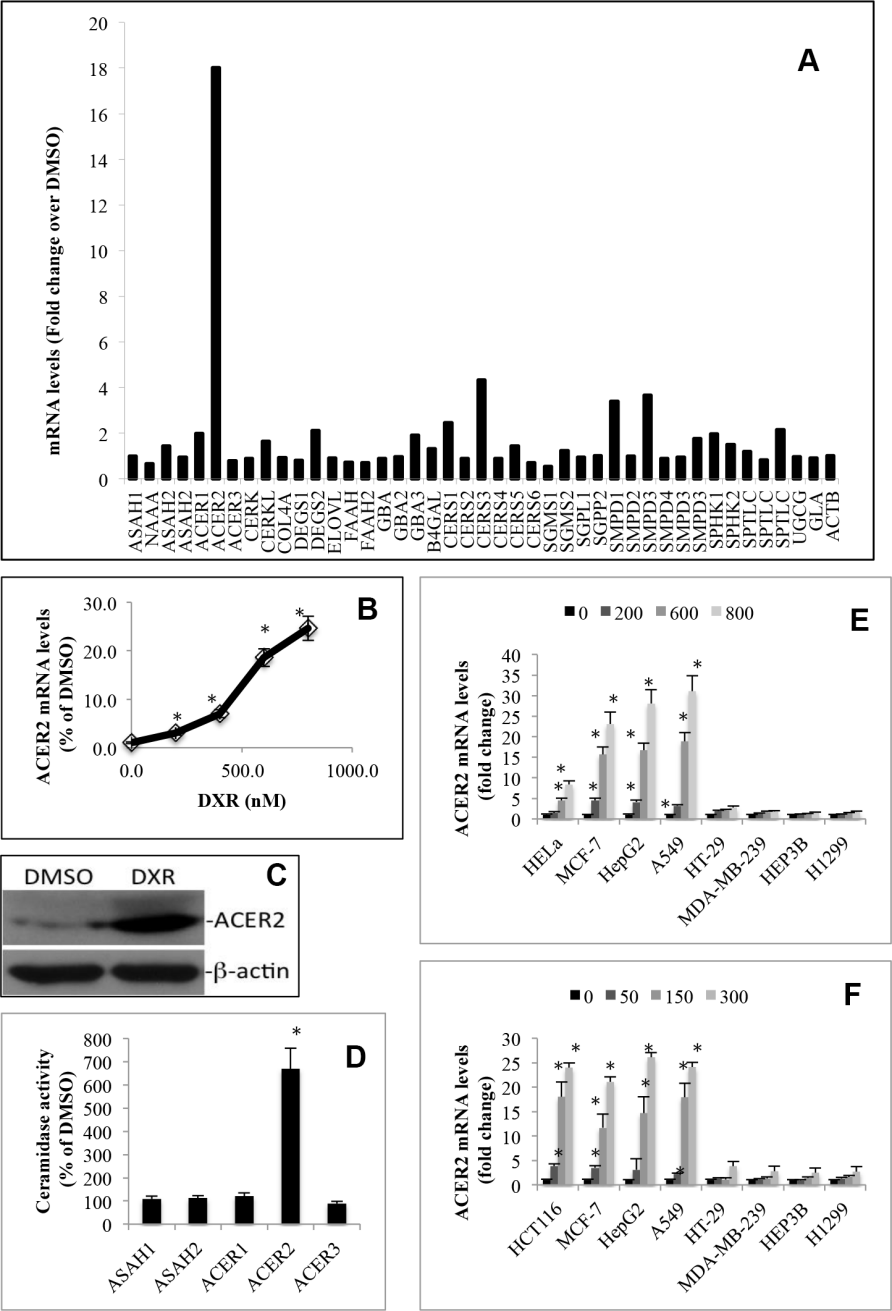
DNA damage upregulates ACER2 in various cell lines HCT116 cells grown to an 80% confluence were treated with DMSO or DXR at 800 nM for 24 h before total RNA was extracted and subjected to the profiling of mRNA levels of sphingolipid-metabolizing enzymes by a PCR array (**A**). HCT116 grown to a 75% confluence were treated with DXR at 200, 400, 600, or 800 nM or DMSO for 24 h before the cells were harvested and subjected to qPCR analyses for quantifying ACER2 mRNA levels (**B**). HCT116 were grown to a 75% confluence and treated with DMSO or DXR at 800 nM for 24 h before ACER2 protein levels were determined by Western blot analyses using anti-ACER2 antibody (**C**). HCT116 grown to an 80% confluence were treated with DXR at 800 nM or DMSO for 24 h. The cells were harvested and subjected to ceramidase activity assays with the indicated ceramides or ceramide analogues as substrates at pH 4.5, pH 7.4, or pH 8.5 as described in Materials and Methods (**D**). HeLa, MCF-7, HepG2, A549, HT-29, MDA-MB-231, HEP3A, or H1299 cells were treated with DMSO or DXR at 200, 400, 600, or 800 nM for 24 h before ACER2 mRNA levels were determined by qPCR (**E**). HCT116, MCF-7, HepG2, A549, HT-29, MDA-MB-231, HEP3A, or H1299 cells were treated with DMSO or 5-FU at 50, 150, or 300 nM for 24 h before ACER2 mRNA levels were determined by qPCR (**F**). Data represent mean values ± SD of 3 independent experiments. Image data represent one of three experiments. *p-values of DXR vs DMSO.

To better understand the molecular mechanism by which DNA damage regulates bioactive sphingolipids, we investigated how DNA damage globally alters the expression of major sphingolipid-metabolizing enzymes (Supplementary Figure S1) by conducting a sphingolipid pathway-specific qPCR array that simultaneously quantifies major sphingolipid-metabolizing enzymes [31]. We demonstrated that treatment with DXR caused a marked increase in the mRNA levels of ACER2, in addition to a moderate increase in the mRNA levels of ceramide-generating enzymes including ceramide synthase 3 (CERS3), acid sphingomyelinase (SMPD1), and neutral sphingomyelinase 2 (SMPD3) without affecting SPHK1 or SPHK2 mRNA levels in HCT116 cells (Figure 2A). qPCR analyses confirmed that DXR increased ACER2 mRNA levels in HCT116 in a dose-dependent manner (Figure 2B). Moreover, DXR also increased ACER2 protein level (Figure 2C) and its enzymatic activity (Figure 2D). We also confirmed that DXR failed to alter ceramidase activity encoded by ASAH1, ASAH2, ACER1, or ACER3 in these cells (Figure 2D).

To determine if ACER2 upregulation by DXR is a cell type-dependent or independent event, we tested if DXR increased ACER2 mRNA levels in other cell lines. Interestingly, treatment with DXR increased ACER2 mRNA levels in HeLa, MCF-7, A459, or HepG2 cells but not in MDA-MB-231, H1299, or Hep3B (Figure 2E). These results suggest that DXR-induced upregulation of ACER2 is a cell type-specific event.

To determine if DXR upregulated ACER2 through DNA damage, we determined if treatment with a different DNA damaging agent upregulated ACER2 in selected cancer cell lines. Indeed, we found that 5-fluorouracil (5-FU), which induces DNA damage through a mechanism different from DXR, also upregulated ACER2 mRNA levels in HCT116, MCF-7, A459, or HepG2 cells but not in HT-29, MDA-MB-231, H1299, or Hep3B cells (Figure 2F), confirming that DNA damage upregulates ACER2 independently of genotoxic stress type.

### ACER2 regulates the levels of bioactive sphingolipids in response to DNA damage

**Figure 3:**
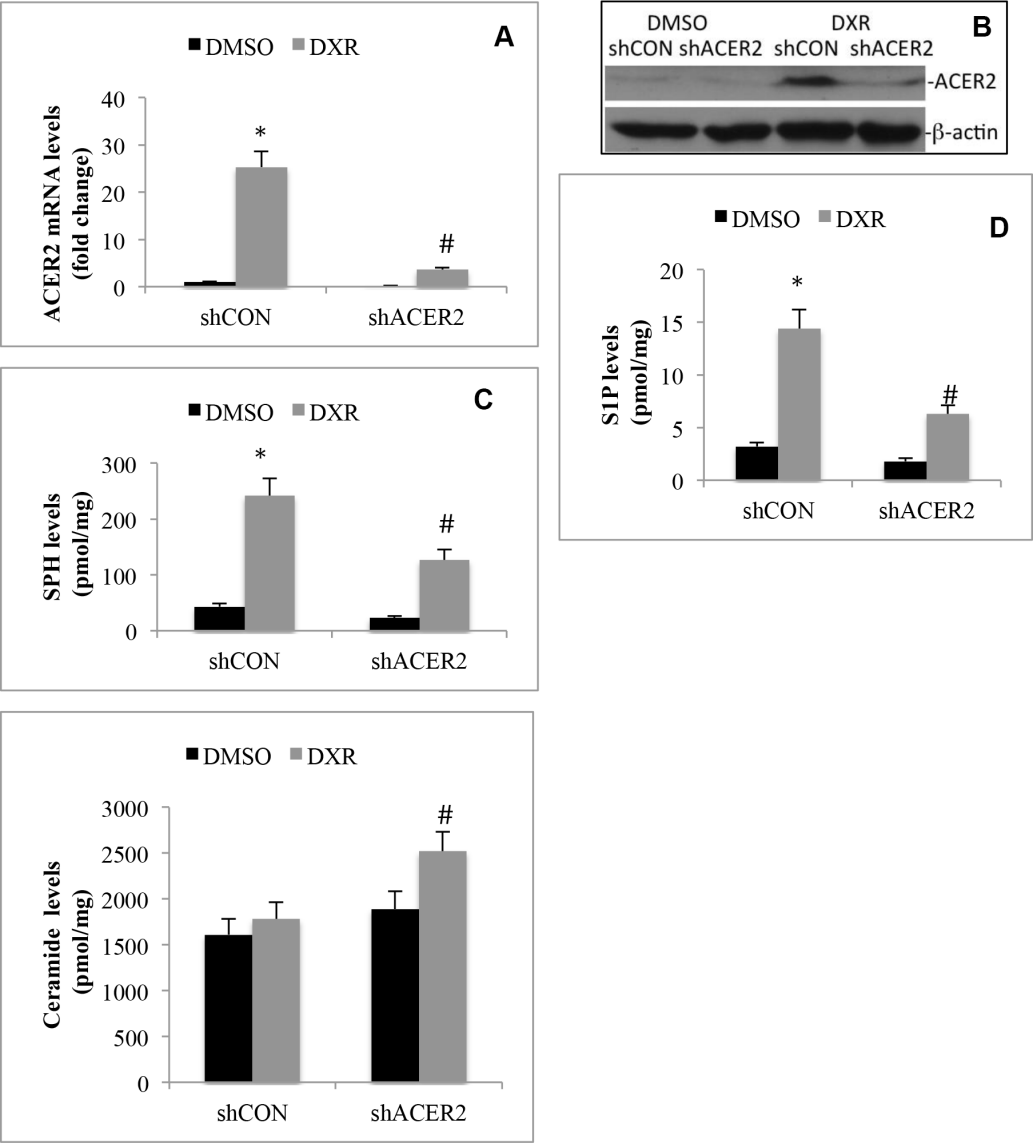
DNA damage increases the generation of SPH and S1P by upregulating ACER2 HCT116 cell lines that stably express ACER2-specific shRNA (shACER2) and a control shRNA (shCON), respectively, were treated with DXR (800 nM) or DMSO for 24 h before ACER2 mRNA levels were measured by qPCR (**A**), ACER2 protein was analyzed by Western blot analyses (**B**), and the levels of SPH (**C**), S1P (**D**), or ceramides (**E**) were analyzed by LC-MS/MS. Data represent mean values ± SD of 3 independent experiments. *p-values of DXR vs DMSO, and #*p*-values of shACER2/DXR vs shCON/DXR.

We previously demonstrated that ACER2 overexpression increases both SPH and S1P levels in T-REX-HeLa cells [30]. This prompted us to test the hypothesis that ACER2 upregulation mediates the increases in the levels of both SPH and S1P in cells in response to DNA damage by investigating if knocking down ACER2 inhibited the DXR-induced increases in the levels of both SPH and S1P in HCT116 cells. We knocked down ACER2 in HCT116 cells through lentiviral expression of an ACER2-specific shRNA, shACER2. DXR increased ACER2 mRNA levels in cells transduced with lentiviruses expressing a control shRNA (shCON-HCT116 cells) but not in cells transduced with lentiviruses expressing shACER2 (shACER2-HCT116 cells) (Figure 3A). Treatment with DXR increased ACER2 protein in shCON-HCT116 cells but not in shACER2-HCT116 cells (Figure 3B). Knocking down ACER2 inhibited DXR-induced increase in the levels of SPH (Figure 3C) or S1P (Figure 3D) while enhancing DXR-induced increase in the levels of ceramides (Figure 3E) in HCT116 cells, suggesting that ACER2 upregulation mediates a significant portion of the increases in both SPH and S1P levels in cells in response to DNA damage. These results also indicate that ceramides generated in response to DNA damage are converted to SPH in part by the action of the upregulated ACER2; thereby explaining why treatment with DXR only slightly increases the levels of ceramides in HCT116 cells although ceramide-producing enzymes are upregulated.

### ACER2 knockdown inhibits programmed cell death in response to DNA damage

**Figure 4:**
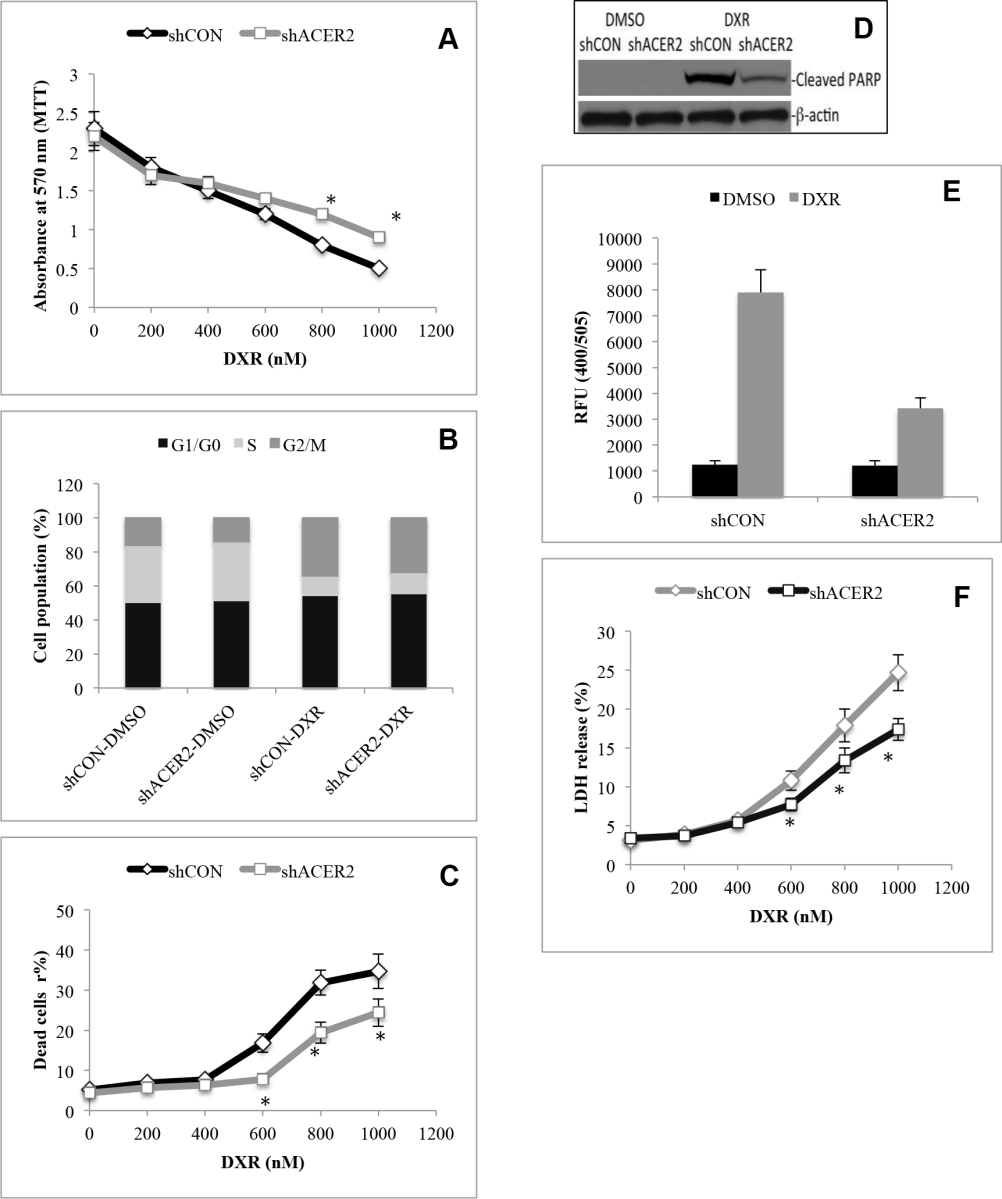
ACER2 knockdown inhibits programmed cell death in response to DNA damage HCT116 cells stably expressing shCON or shACER2 were treated with DXR at 200, 400, 600, 800, 1000 nM or DMSO for 48 h before being subjected to MTT assays (**A**). HCT116 cells stably expressing shCON or shACER2 were treated with DXR at 400 nM or DMSO for 48 h before being subjected to cell cycle analyses by flow cytometry (**B**). HCT116 cells stably expressing shCON or shACER2 were treated with DXR at 200, 400, 600, 800, 1000 nM or DMSO for 48 h before being subjected to PI/AA co-staining (**C**). HCT116 cells stably expressing shCON or shACER2 were treated with DXR at 800 nM or DMSO for 48 h before Western blot analyses for PARP cleavage (**D**) or caspase 3 activity assays (**E**). HCT116 cells stably expressing shCON or shACER2 were treated with DXR at 200, 400, 600, 800, 1000 nM or DMSO for 48 h before being subjected to LDH release assays (**F**). Data represent mean values ± SD of 3 independent experiments. *p-values of shACER2 vs shCON.

To test whether ACER2 upregulation was involved in PCD in response to DNA damage, we determined if ACER2 knockdown affected PCD in HCT116 cells in response to DXR. shCON-HCT116 cells or shACER2-HCT116 cells were treated with different concentrations of DXR. MTT assays showed that knocking down ACER2 inhibited the reduction in the viable cell population in response to treatment with DXR at high concentrations (≥ 600 nM) but not low concentrations (≤ 400 nM) (Figure 4A). Treatment with DXR at a low concentration (400 nM) induced cell cycle arrest at the G2/M phase to a similar extent in shCON-HCT116 or shACER2-HCT116 cells (Figure 4B and Supplementary Figure S2A) without increasing the cell population (PI^+^ and/or AA^+^) stained positively by propidium iodide (PI) and/or Alexa Fluor 488-Annexin V (AA), PARP cleavage, or LDH release from either cell line (Data not shown), suggesting that a moderate upregulation of ACER2 does not affect the anti-proliferative effects induced by DXR at low concentrations. Treatment with DXR at a high concentration (1,000 nM) induced a marked increase in the PI^+^ and/or AA^+^ cell population (Figure 4C and Supplementary Figure S2B), PARP cleavage (Figure 4D), and caspase 3/7 activity (Figure 4E) in HCT116 cells as well as LDH release from these cells (Figure 4F), and these effects were inhibited by ACER2 knockdown (Figure 4C, 4D, 4E, and 4F), suggesting that knocking down ACER2 inhibits PCD in HCT116 cells in response to DXR at high concentrations. Because the concentration of DXR correlates with the extent of DNA damage [32], high concentrations of DXR caused more DNA damage than low concentrations of DXR. Based on these observations, we conclude that ACER2 upregulation mediates at least part of the PCD in response to a high degree of DNA damage.

To further validate this notion, we determined if ACER2 upregulation also mediated PCD in response to a different DNA damaging agent, 5-FU. shCON-HCT116 or shACER2-HCT116 cells were treated with 5-FU or DMSO for 48 h before PCD was assessed as described earlier. We found that ACER2 knockdown inhibited a decrease in the viable cell population measured by MTT assays (Supplementary Figure S3A), an increase in the apoptotic and/or necrotic cell population measured by PI/AA co-staining (Supplementary Figure S3B), and an increase in LDH release (Supplementary Figure S3C) in response to treatment with 5-FU. We also confirmed that ACER2 knockdown inhibited 5-FU-induced increase in the levels of SPH (Supplementary Figure S3D) or S1P (Supplementary Figure S3E). These results support the notion that ACER2 upregulation mediates PCD in response to DNA damage.

### ACER2 overexpression induces PCD

**Figure 5:**
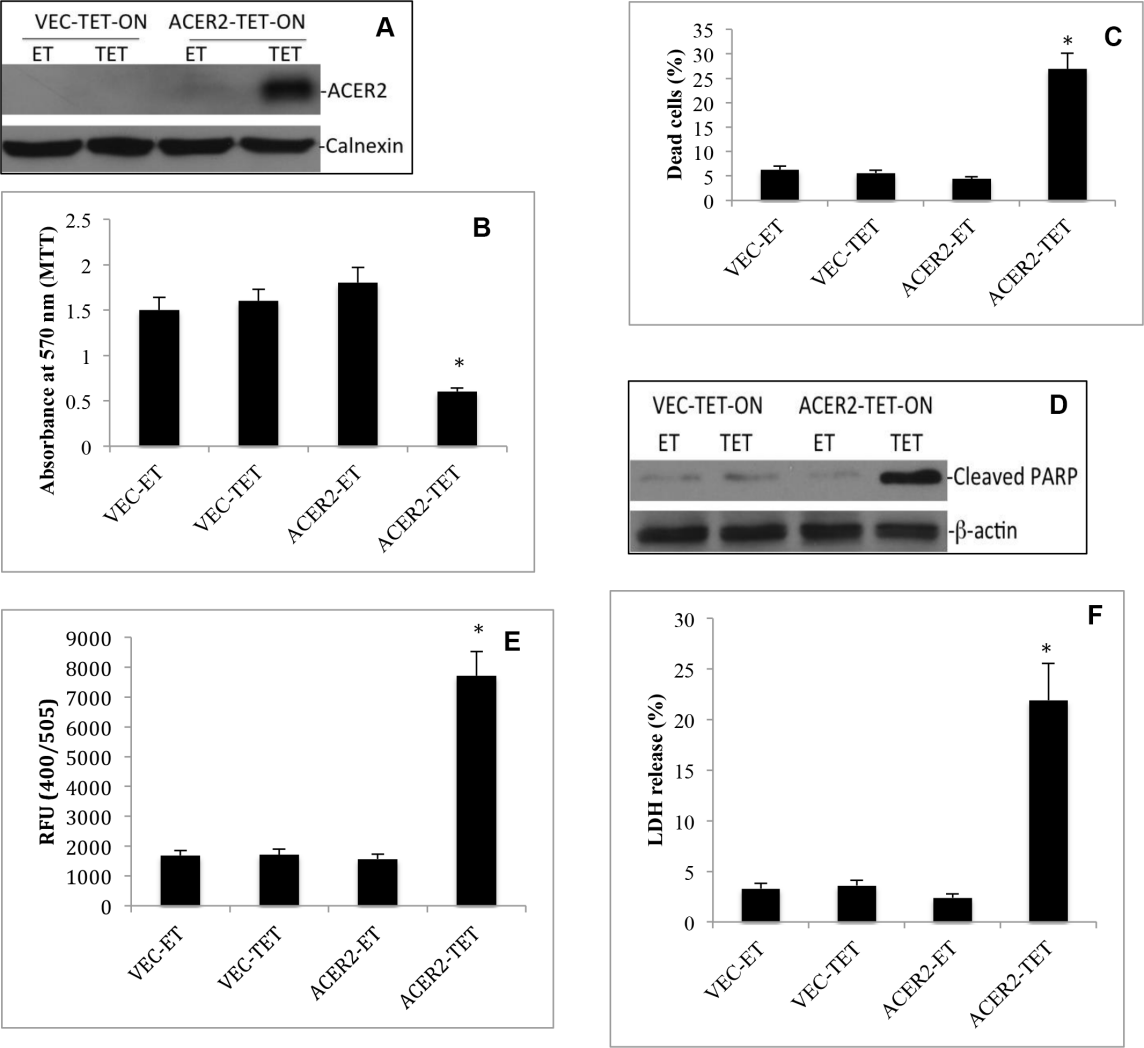
High but not low ectopic expression of ACER2 induces programmed cell death in T-REx™-HeLa cells VEC-TET-ON or haCER2-TET-ON cells were grown to a 25% confluence before they were treated with TET (20 ng/ml) or ET. At 72 h post treatment with ET or TET, the cells were subjected to Western blot analyses using anti-ACER2 antibody (**A**), MTT assays (**B**), PI/AA co-staining assays (**C**), Western blot analyses for PARP cleavage (**D**), caspase 3 activity assays (**E**), and LDH release assays (**F**). Image data represent one of three experiments. Numeral data represent mean values ± SD of 3 independent experiments. *p-values of ACER2-TET vs ACER2-ET.

Following the above findings, we determined if ectopic expression of ACER2 at a high level but not low level mimicked DXR to induce PCD in cells. To ectopically express ACER2 in a regulated manner, we used the ACER2 overexpression cell line haCER2-TET-ON that we previously generated [30] and an isogenic control cell line, Vec-TET-ON. The haCER2-TET-ON cell line is a T-REX-HeLa derivative that ectopically expresses ACER2 under the control of a tetracycline-regulated promoter system, CMV-TET-ON [30], and Vec-TET-ON is an isogenic cell line which was stably transfected with an empty TET-ON vector. In the presence of ethanol (ET), the vehicle of tetracycline (TET), ACER2 expression was moderately increased in haCER2-TET-ON cells compared to VEC-TET-ON cells due to a leakiness of the CMV-TET-ON promoter system (Figure 5A). This moderate upregulation of ACER2 did not affect cell viability (Figure 5B), the PI^+^/AA^+^ cell population (Figure 5B), PARP cleavage (Figure 5C), or LDH release (Figure 5E). Upon treatment with TET, ACER2 expression was significantly increased in haCER2-TET-ON cells but not in VEC-TET-ON cells (Figure 5A). Treatment with TET caused a reduction in the viable cell population (Figure 5B), an increase in the PI^+^/AA^+^ cell population (Figure 5C and Supplementary Figure S2C), PARP cleavage (Figure 5D), and an increase in caspase 3 activity (Figure 5E) in haCER2-TET-ON cells but not in VEC-TET-ON cells. Consistently, treatment with TET also induced LDH release from haCER2-TET-ON cells but not VEC-TET-ON cells (Figure 5F). These results demonstrate that high upregulation of ACER2 is sufficient to induce PCD in cells, and they are consistent with the role of endogenous ACER2 in mediating PCD in response to DNA damage.

### ACER2 upregulation induces PCD through SPH

**Figure 6:**
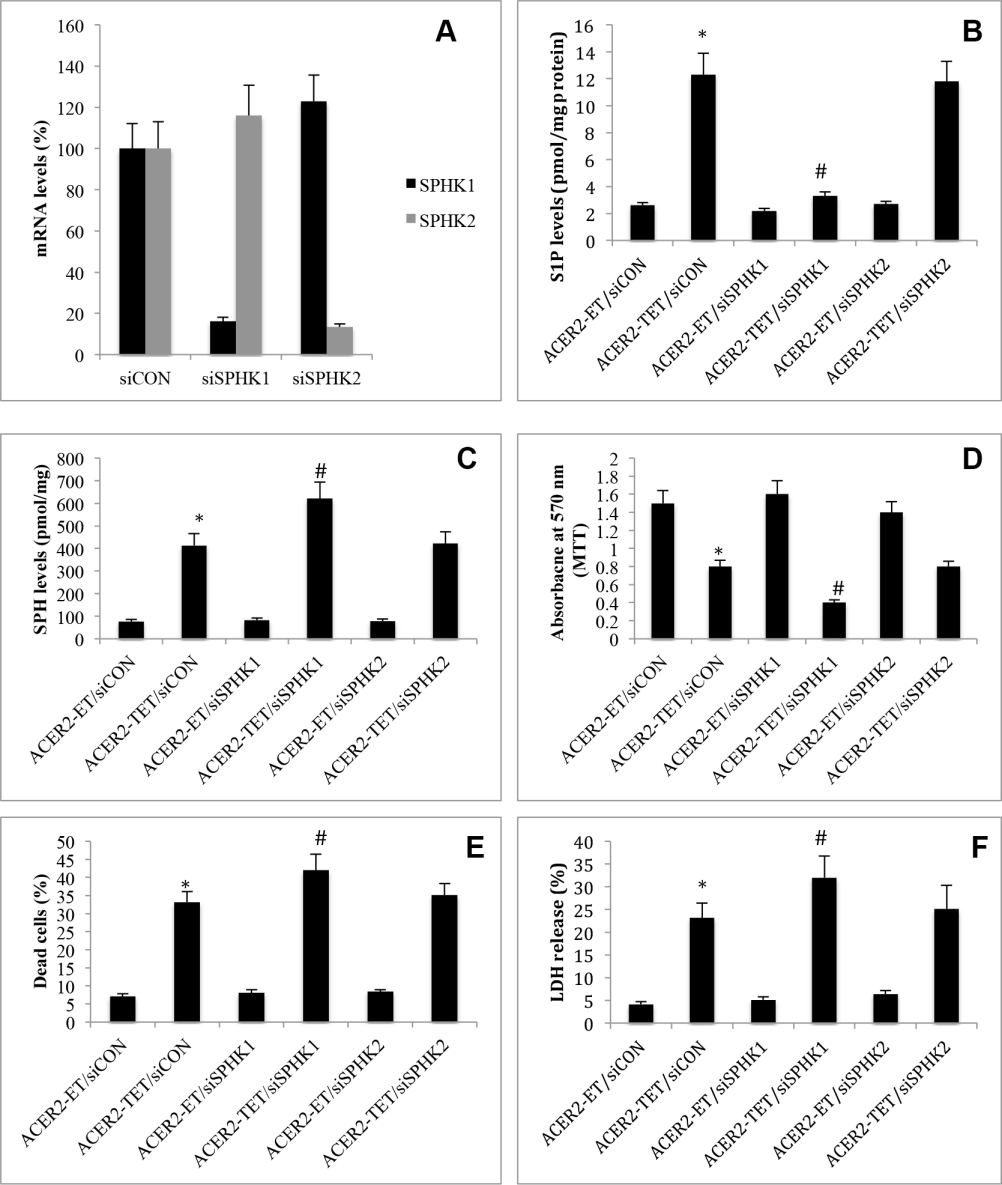
High ectopic expression of ACER2 induces programmed cell death through SPH haCER2-TET-ON cells at a 25% confluence were transfected with siCON, siSPHK1, or siSPHK2 for 48 h before SPHK1 or SPHK2 mRNA levels were determined by qPCR (**A**). haCER2-TET-ON cells at a 25% confluence were transfected with siCON, siSPHK1, or siSPHK2 for 24 h before they were treated with ET or TET (20 ng/ml) for 72 h. Post ET or TET treatment, the cells were subjected to LC-MS/MS analyses for the levels of S1P (**B**) and SPH (**C**), MTT assays (**D**), PI/AA co-staining (**E**), or LDH release assays (**F**). Data represent mean values ± SD of 3 independent experiments. *p-values of ACER2-TET/siCON vs ACER2-ET/siCON; and #*p*-values of ACER2-TET/siSPHK1 vs ACER2-TET/siCON.

Because ACER2 upregulation increases the levels of both SPH and S1P, with both having been implicated in PCD [21], we determined which bioactive lipid is the lipid mediator of ACER2 in PCD by testing if blocking the conversion of SPH to S1P enhanced or attenuated PCD in response to ACER2 overexpression. Because SPH is phosphorylated by SPHK1 and SPHK2, we knocked down SPHK1 or SPHK2 by RNA interference (RNAi) in haCER2-TET-ON cells. A small interfering RNA (siRNA) against SPHK1 (siSPHK1) or SPHK2 (siSPHK2), or a control siRNAs (siCON) was transfected into haCER2-TET-ON before ACER2 overexpression was induced. qPCR analyses showed that transfection with siSPHK1 and siSPHK2 decreased the mRNA levels of SPHK1 and SPHK2, respectively, compared to transfection with siCON in haCER2-TET-ON cells (Figure 6A). Knocking down SPHK1 but not SPHK2 markedly inhibited the increase in the levels of S1P (Figure 6B) while enhancing the increase in the levels of SPH (Figure 6C) in haCER2-TET-ON cells in response to ACER2 overexpression. Knocking down SPHK1 but not SPHK2 also enhanced the reduction in cell viability (Figure 6D) and the increase in the PI^+^/AA^+^ cell population (Figure 6E) in haCER2-TET-ON cells in response to ACER2 overexpression as well as LDH release from these cells (Figure 6F) in response to overexpression of ACER2. These results indicate that inhibiting SPHK1 enhances PCD in response to ACER2 overexpression. To confirm this notion, we investigated if treatment with PF-543, a highly specific and potent inhibitor of SPHK1 [33], also enhanced PCD in response to ACER2 overexpression. haCER2-TET-ON cells were treated with PF-543 or the vehicle DMSO before ACER2 overexpression was induced. Similar to RNAi-mediated knockdown of SPHK1, treatment with PF-543 nearly abolished the increase in S1P levels (Supplementary Figure S3A) while enhancing the increase in the levels of SPH in haCER2-TET-ON cells in response to overexpression of ACER2 (Supplementary Figure S3B), confirming that the conversion of SPH to S1P is mainly mediated by SPHK1 in these cells. Treatment with PF-543 enhanced the viability reduction (Supplementary Figure S3C), the increase in the number of PI^+^/AA+ cells (Supplementary Figure S3D) in haCER2-TET-ON cells as well as LDH release from these cells (Supplementary Figure S3E) in response to overexpression of ACER2. These results confirm that inhibiting the SPHK1-mediated conversion of SPH to S1P enhances PCD in response to ACER2 upregulation and suggests that SPH but not S1P or its metabolites is the lipid mediator of ACER2 in PCD. Taken together, these results suggest that SPH but not S1P or its metabolites is the lipid mediator of ACER2 in PCD.

### Ceramides indirectly mediate PCD in response to DNA damage as the substrates of ACER2

**Figure 7:**
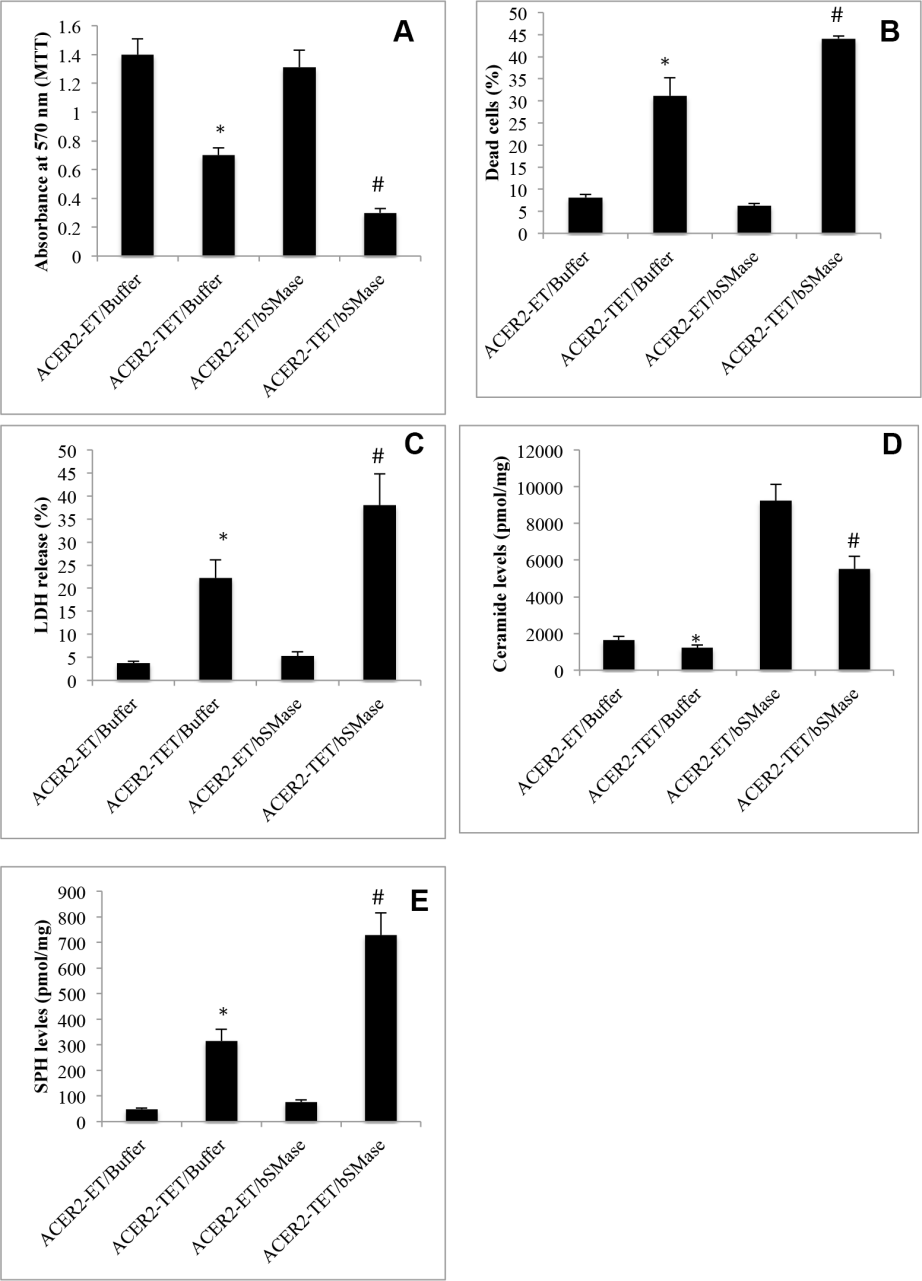
Increasing the generation of ceramides enhances programmed cell death in response to ACER2 overexpression haCER2-TET-ON cells were grown in the presence of ET/Buffer, TET (20 ng/ml)/Buffer, ET/bSMase (0.2 U/ml), or TET/bSMase for 3 days before MTT assays (**A**), PI/AA co-staining (**B**), LDH release assays (**C**), and LC-MS/MS analyses for the levels of SPH (**D**) and ceramides (**E**). Data represent mean values ± SD of 3 independent experiments. *p-values of ACER2-TET/Buffer vs ACER2-ET/Buffer; and #*p*-values of ACER2-TET/bSMase vs ACER2-TET/Buffer.

Many studies demonstrated that DNA damage increases the levels of ceramides and that increased ceramides directly mediate PCD in response to DNA damage [4–12]. However, our previous study indicated that their metabolite SPH but not ceramides in the Golgi complex (referred to as Golgi ceramides) *per se* directly mediates PCD in cells [34]. If this hypothesis is correct, increasing the levels of Golgi ceramides should enhance PCD in response to ACER2 upregulation. To test this hypothesis, we determined if treatment with bacterial sphingomyelinase (bSMase) enhanced PCD in haCER2-TET-ON cells in response to ACER2 overexpression. We previously demonstrated that treatment with bSMase, which releases ceramides from sphingomyelins on the plasma membrane, enhanced the generation of both SPH and S1P in HeLa cells in response to ACER2 overexpression [30], suggesting that ceramides generated on the plasma membrane are transported to the Golgi complex where they are hydrolyzed into SPH by ACER2. Expectedly, treatment with bSMase only slightly induced a reduction in the viable cell number (Figure 7A) and an increase in the PI^+^/AA^+^ cell population (Figure 7B) in haCER2-TET-ON cells and LDH release from these cells (Figure 7C) when ACER2 overexpression was not induced. However, treatment with bSMase significantly enhanced the reduction in the viable cell number (Figure 7A) and the increase in the PI^+^/AA^+^ cell population (Figure 7B) in haCER2-TET-ON cells as well as LDH release from these cells (Figure 7C) in response to ACER2 overexpression. LC-MS/MS confirmed that treatment with bSMase caused a marked increase in the levels of ceramides (Figure 7D) but a slight increase in the levels of SPH (Figure 7E) in haCER2-TET-ON cells when ACER2 overexpresson was not induced. Upon ACER2 overexpression, the bSMase-induced increase in the levels of SPH was markedly enhanced (Figure 7E) whereas the bSMase-induced increase in the levels of ceramides was significantly attenuated in haCER2-TET-ON cells (Figure 7D). Taken together, these results suggest that Golgi ceramides that serve as the substrates of ACER2 do not directly mediate PCD although their increase enhances the ability of ACER2 to induce PCD by increasing the production of SPH. These results support the notion that ACER2 upregulation mediates PCD in response to DNA damage by converting DNA-damage-induced ceramides into SPH and suggest that high degree of DNA damage maximizes the production of SPH and SPH-mediated PCD by upregulating both ACER2 and its substrates.

### Upregulation of the ACER2/SPH pathway induces programmed cell death by inducing ROS production

**Figure 8:**
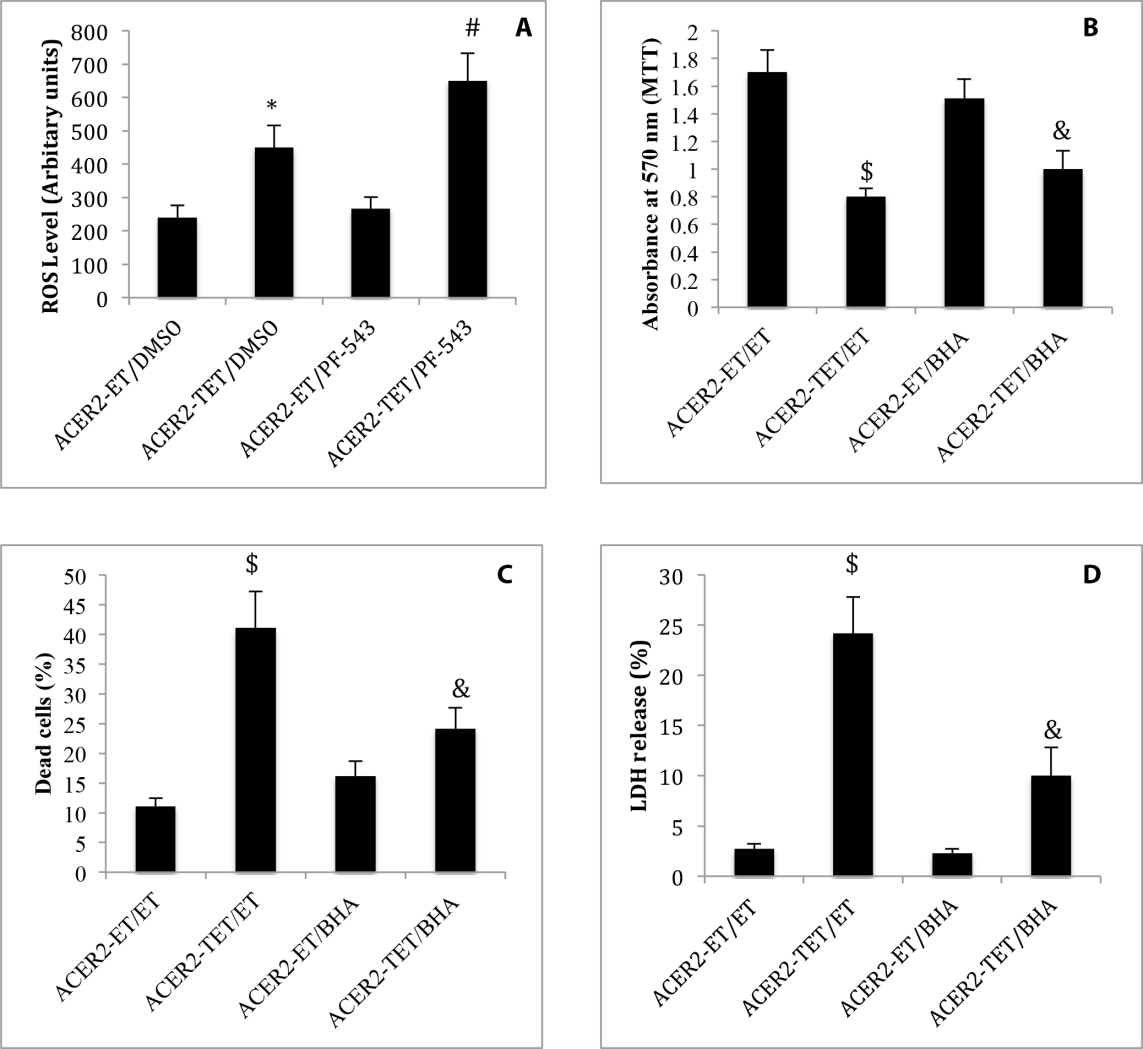
ACER2 overexpression induces ROS production haCER2-TET-ON cells grown in the presence of ET/DMSO, TET (20 ng/ml)/DMSO, ET/PF-543 (100 nM), or TET/PF-543 for 48 h before ROS levels were assayed (**A**). haCER2-TET-ON cells were pre-treated with ET or BHA (100 μM) for 2 h before being treated with ET or TET (20 ng/ml) for 48 h. Cells were subjected to MTT assays (**B**), PI/AA co-staining (**C**), and LDH release assays (**D**). Data represent mean values ± SD of 3 independent experiments. *p-values of ACER2-TET/DMSO vs ACER2-ET/DMSO; #*p*-values of ACER2-TET/PF543 vs ACER2-TET/DMSO; $*p*-values of ACER2-TET/ET; and and *p*-values of ACER2-TET/BHA vs ACER2-TET/ET.

Previous studies demonstrated that SPH and its analogs, when added exogenously to cells, induce the production of reactive oxygen species (ROS) in various cell types [35–37], suggesting that endogenous SPH generated by the action of ACER2 may induce ROS in cells. To test this, we determined if ectopic expression of ACER2 induced ROS production by increasing SPH in HaCER2-TET-ON cells. haCER2-TET-ON cells were treated with the SPHK1 inhibitor PF-543 or the vehicle DMSO before ACER2 overexpression was induced by TET. ROS assays found that ACER2 overexpression increased ROS levels in haCER2-TET-ON cells, and this was further enhanced by treatment with PF-543 (Figure 8A), suggesting that ACER2 upregulation indeed induces ROS production through SPH. Following this finding, we then determined if ROS production plays a role in mediating PCD in response to the upregulation of the ACER2/SPH pathway. We demonstrated that treatment with the radical scavenger butylated hydroxyanisole (BHA) markedly inhibited the reduction in the viable cell number (Figure 8B) and the increase in the PI^+^/AA^+^ cell population (Figure 8C) in haCER2-TET-ON cells and LDH release from these cells (Figure 8D) in response to ACER2 overexpression. Taken together, these results suggest that upregualtion of the ACER2/SPH pathway mediates PCD in response to DNA damage at least in part by increasing ROS production.

## DISCUSSION

In this study, we for the first time reveal that different degrees of DNA damage upregulate the expression of ACER2 to different levels and that high but not low upregulation of ACER2 mediates PCD in response to DNA damage by accumulating SPH, a pro-death bioactive lipid that induces the production of ROS. This study highlights the role of ACER2 and its regulated bioactive lipid SPH in the DDR.

ACER2 is a member in the alkaline ceramidase family that we previously identified [24, 30]. ACER2 has seven putative transmembrane domains and is localized to the Golgi complex [30, 38]. We previously found that ACER2 was upregulated in human tumor cells in response to various forms of stress including serum deprivation [30] and treatment with the retinoid 4-N-hydroxyphenyl retinamide (4-HPR) [39]. In this current study, we showed that treatment with the DNA damaging agent DXR or 5-FU upregulated ACER2 in many but not all cancer cell lines that we examined (Figure 2). Of note, DXR or 5-FU upregualted ACER2 in the cell lines that express wild-type p53 but not in the cell lines that express mutant p53 or lack p53. Sequence analyses found a putative p53 responsive element in the first intron of the ACER2 gene that perfectly matches the consensus sequence of p53 binding sites, indicating that ACER2 may be a transcriptional target of p53. Indeed, our unpublished results confirmed that ACER2 is a direct transcriptional target of p53 (manuscript in preparation). These results suggest that DNA damage upregulates ACER2 in tumor cells by activating p53. In addition to ACER2, several other ceramidase genes have been identified in mammalian cells [24]. Previous studies by other groups demonstrated that DNA damaging agents also increase the expression of the acid ceramidase gene (ASHA1) [27, 40, 41] or transiently increase its activity in certain cancer cell lines through a post-translational modification [26] whereas Uchida et al. [29] showed that DNA damage by ultraviolet radiation (UVR) decreases ASHA1 and ASHA2 (the neutral ceramidase) activity in epidermal keratinocytes. However, we found that neither the expression of ASAH1 and ASAH2 nor their activity was increased in HCT116 cells, suggesting that DNA damage regulates ACER2 and other ceramidases through different mechanisms.

We previously demonstrated that ACER2 catalyzes the hydrolysis of various ceramide species into SPH in a Ca^2+^-dependent manner both *in vitro* and *in vivo* [38], so its upregulation would lead to an increase in the levels of SPH and its phosphate S1P. Indeed, we previously demonstrated that ACER2 upregulation increases the levels of both SPH and S1P in T-REX-HeLa cells in response to serum deprivation [30]. In this current study, we demonstrated that ACER2 upregulation mediates the increases in the levels of both SPH and S1P in HCT116 cells in response to the DNA damaging agent DXR (Figure 3) or 5-FU (Supplementary Figure S2). Moreover, Cuvillier *et al.* [15] demonstrated that treatment with DXR increased the levels of SPH in MCF-7 cells although it was unclear which ceramidase was responsible for the SPH rise. Because we showed that DXR also markedly upregulated ACER2 in MCF-7 cells, it is conceivable that DXR increases the levels of SPH likely by upregulating ACER2 in these cells. Taken together, these results suggest that DNA damage upregulates ACER2 and its product SPH in various cell lines. Gude et al. [16] demonstrated that DNA damage by DXR increased the levels of S1P in Jurkat T cells and U937 cells by increasing the expression of SPHK1. In HCT116 cells, however, we found that the DXR-induced S1P rise was caused by the upregulation of ACER2 but not SPHK1 or SPHK2 (Figure 2 and Figure 3). These results suggest that ACER2 plays a key role in regulating the generation of both SPH and S1P in cells in response to various forms of stress, including DNA damage, highlighting the role for ACER2 and bioactive sphingolipids regulated by ACER2 in stress responses.

Previous studies showed that SPH was upregulated in human cells in response to different stressful insults, such as glucocorticoid [42], serum deprivation [43], or oxidative stress [36]and that inhibiting the SPH increase with the alkaline ceramidase inhibitor (1*S*,2*R*)-D*erythro*-2-(*N*-myristoylamino)-1-phenyl-1-propanol (D-*e*-MAPP) inhibits PCD in response to these stressful insults. These studies indicated but have not proven that SPH generated by the action of an alkaline ceramidase directly mediates PCD in response to stress because SPH can be further converted to S1P and then hexadecenal, which both have recently been implicated in PCD as well [21]. In this study, we showed that blocking the conversion of SPH to S1P augmented PCD in response to ACER2 overexpression (Figure 6). This result undoubtedly validates that SPH generated by the action of ACER2 is a direct mediator of PCD. We showed that DXR at high concentrations markedly upregulated both ACERR2 and its product SPH (Figure 3) and that knocking down ACER2 inhibited not only the increase in the levels of SPH (Figure 3) but also PCD (Figure 4) in response to treatment with this DNA damaging agent. Moreover, our results also demonstrated that ACER2 upregulation mediates PCD in HCT116 cells in response to 5-FU (Supplementary Figure S2). Taken together, these results suggest that a high degree of DNA damage induces PCD at least in part by upregulating the ACER2/SPH pathway. We previously demonstrated that ACER2 can also be upregulated by the retinoid 4-HPR in SCC14 head and neck cancer cells and that its upregulation mediates the 4-HPR-induced generation of dihydrosphingosine (DHS), which in turns induces the death of these cells [39]. These results suggest that ACER2 regulates PCD through different sphingoid bases in response to different stressful insults.

Previous studies demonstrated that inhibiting SPHK1 sensitized cancer cells to PCD in response to DNA damage [44–46], suggesting that increased S1P may protect cells from PCD in response to DNA damage. Because ACER2 upregulation increases the levels of S1P, in addition to SPH, its upegulation could promote cell survival in certain contexts. Indeed, we previously demonstrated that a moderate upregulation of ACER2 increases the levels of S1P in T-REX HeLa cells without accumulating SPH to a cytotoxic level, thereby promoting cell survival under the stress of serum deprivation [47]. In this current study, we showed that a moderate upregulation of ACER2 in response to a low level of DNA damage increases the levels of S1P without accumulating SPH in cells (Figure 1 and Figure 3). The increased S1P may override the cytotoxicity of a low level of SPH. This may explain why the low upregulation of ACER2 did not induce PCD in response to a low degree of DNA damage.

Numerous studies demonstrated that increased ceramides mediate PCD in response to various forms of stress, including DNA damage [4–12]. However, our previous studies indicated that Golgi ceramides indirectly mediate PCD likely through their metabolite SPH [34]. This view is supported by several lines of evidence present in this current study. First, knocking down ACER2 did not induce PCD in HCT116 cells although ceramides were elevated (Figure 4). Second, we showed that treatment with bSMase did not induce PCD in cells expressing a low basal level of ACER2 (Figure 7) although this treatment caused a marked increase in the cellular levels of ceramides [48] (Figure 7). Third, treatment with bSMase markedly enhanced PCD in cells in response to ACER2 overexpression although bSMase-induced increase in the levels of ceramides was attenuated by ACER2 overexpression (Figure 7). The stimulatory effect of bSMase on PCD in response to ACER2 upregulation is likely due to an enhancement in the increase in the levels of SPH because bSMase treatment caused a slight increase in the levels of SPH in cells expressing a low level of ACER2, but did so markedly upon ACER2 expression induction [48] (Figure 7). These results clearly suggest that Golgi ceramides do not directly induce PCD whereas their metabolite SPH generated by the action of ACER2 does. However, as the substrates of ACER2, Golgi ceramides are necessary for the ACER2-mediated generation of SPH, and their increase can further enhance the generation of SPH and PCD in response to ACER2 upregulation. From these observations, we conclude that a high degree of DNA damage maximizes the production of SPH by upregulating both ACER2 and its cellular substrates, ceramides, to ensure PCD execution in cells with irreparable damaged genomes.

SPH has been shown to activate various signaling pathways implicated in PCD, including ROS production [46]. Similar to SPH, several SPH analogs also induced ROS production in different cell types [35–37]. However, in all these studies, exogenous SPH was used, so it remains unclear if endogenous SPH has such an effect. In this current study, to our knowledge, we for the first time demonstrated that increasing endogenous SPH due to ACER2 upregulation induced ROS production (Figure 8). More importantly, we proved that the increased production of ROS mediates PCD induced by ACER2 upregulation (Figure 8). How ACER2-generated SPH increases the cellular ROS levels remains unclear. Zigdon *et al.* [49] demonstrated that an accumulation of DHS, a saturated analog of SPH, due to a deficiency of (dihydro)ceramide synthase 2 (CerS2) increases ROS levels in liver cells by disrupting the mitochondrial respiratory chain. Whether ACER2-generated SPH increases ROS levels through the same mechanism is currently under investigations in our laboratory.

In conclusion, our results present in this study demonstrate that ACER2 is upregulated in p53-proficient cancer cell lines but not p53-defieint cell lines in response to DNA damage and that ACER2 upregulation mediates PCD in response to a high degree of DNA damage by accumulating SPH, a bioactive lipid that in turn induces cellular ROS production. Moreover, we demonstrate that either inhibiting the conversion of SPH to S1P or increasing the generation of ceramides markedly enhances the ability of the ACER2/SPH pathway to induce PCD in cells. Based on these novel and important findings, we envision that ACER2 activation in combination with SPHK inhibition and ceramide induction represents a novel modality in cancer prevention and therapy.

## MATERIALS AND METHODS

### Reagents

The anti-ACER2 antibody was raised against a peptide located at the carboxyl terminal of ACER2 in our laboratory [30]. Anti-calnexin antibody was purchased from BD Biosciences (San Jose, CA, USA), antibodies against poly (ADP-ribose) polymerase (PARP), cleaved caspase 3, and caspase 9 from Cell Signaling Technology (Danvers, MA, USA). Minimal Essential Medium (MEM), fetal bovine serum (FBS), trypsin-EDTA, Ca^2+^-free phosphate buffered saline (PBS), penicillin/streptomycin solution, blasticidin, Zeocin, and G418 were purchased from Invitrogen Corporation (Carlsbad, CA, USA). All sphingolipids and the fluorescent sphingolipid analogs used in this study were purchased from Avanti Polar Lipids (Alabaster, AL, USA). Doxorubicin, 5-FU, puromycin, cycloheximide, anti-β actin antibody, anti-FLAG antibody, *Staphylococcus aureus* SMase, and other unlisted chemicals were purchased from Sigma (St. Louis, MO, USA).

### Cell culture

HEK293T, HCT116, HeLa, HT-29, MCF-7, MDA-MB-231, A459, H1299, HepG2, and HEP3A were cultured in DMEM or MEM supplemented with 10% FBS and a 1% penicillin/streptomycin solution (the standard supplement). T-REx™-HeLa cells, a HeLa derivative that stably expresses the tetracycline repressor protein, were cultured in MEM supplemented with 5 μg/ml blasticidin in addition to the standard supplement. HaCER2-TET-ON cells, a T-REx™-HeLa derivative that overexpresses ACER2 under the control of a TET-ON system was generated in our previous studies [30]. VEC-TET-ON cells, another T-REx™-HeLa derivative which was stably transfected with the empty TET-ON vector, was also generated in our previous study. Both TET-ON cell lines were cultured in MEM supplemented with blasticidin (5 μg/ml) and Zeocin (25 μg/ml) in addition to the standard supplement.

### RNA interference (RNAi) by small interference RNA (siRNA)

A control siRNA [siCON; 5′-UAAGGCUAUGAAGA GAUACUU -3′ (sense)/5′-GUAUCUCUUCAUAGCCUU AUU-3′ (anti-sense)], SPHK1-specific siRNA [siSPHK1; 5′-AAGGGCAAGGCCUUGCAGCUCUU-3′ (sense)/5′-G AGCUGCAAGGCCUUGCCCUUUU-3′ (antisense)] or SPHK2-specfic siRNA [siSPHK2; 5′-AACGCUUUGCC CUCACCCUUAUU-3′ (sense)/5′-UAAGGGUGAGGG CAAAGCGUUUU] were synthesized in Dharmacon, Inc. (Chicago, IL). siRNA transfection was performed with Oligofectamine (Invitrogen) as described in our previous study [50].

### RNAi by lentiviral expression of shRNA

Lentiviral vectors expressing an ACER2-specific shRNA (shACER2) and a control shRNA (shCON), respectively, were purchased from GeneCopoeia (Rockville, MD, USA). These vectors were packaged into lentiviral particles using HEK293T as host cells according to the manufacturer's instructions. HCT116 cells were transduced with lentiviruses expressing shACER2 or shRNA (shCON). To select cell lines that stably express shACER2 or shCON, the transduced cells were grown in puromycin-containing medium. Puromycin-resistant clones expressing shACER2 or shCON were screened by qPCR analysis for ACER2 mRNA levels and maintained in puromycin-containing medium.

### Quantitative polymerase chain reaction (qPCR)

Total RNA was isolated from cells using RNeasy kits (Qiagen) according to the manufacturer's instructions and qPCR analyses were performed with primer pairs specific ACER2 (5′-AGTGTCCTGTCTGCGGTTACG-3′/5′-TGTTGTTGATGGCAGGCTTGAC-3′) or b-actin (5′-CAATGTTCGGTGCAATTCAGAG-3′/5′-GGATCCC ATTCCTACCACTGTG-3′) as described in our previous studies [39]. qPCR results were analyzed using Q-Gene software which expresses data as mean normalized expression (MNE) [51]. MNE is directly proportional to the amount of mRNA of a target gene relative to the amount of mRNA of the reference gene (β-actin).

### qPCR array

mRNA levels of major sphingolipid metabolizing enzymes were profiled by a sphingolipid pathway-specific qPCR array as described [31].

### LC-MS/MS analysis for sphingolipids

Sphingolipids were analyzed by liquid chromatography tandem mass spectrometry (LC-MS/MS) as described (24) in the Lipidomics Core Facility at Stony Brook University. Briefly, cells were harvested after being washed with ice-cold 25 mM Tris-HCl buffer (pH 7.4) containing 150 mM NaCl. Fifty μl of a mixture (1 μM) of internal sphingolipid standards (IS) including C17SPH, C17SPH-1-phosphate, D-e-C_16_-ceramide (d17:1/16:0), and D-e-_18_-ceramide (d17:1/18:0), was added to each cell pellet sample before lipid extraction with 4 ml of the ethyl acetate/iso-propanol/water (60/30/10%;v/v) solvent system. After centrifugation, one ml of lipid extracts from each sample was used for determination of total phospholipids, and the remaining was used for LC-MS/MS. The lipid extracts from each sample were dried under a stream of nitrogen gas, dissolved in 100 μl of acidified (0.2% formic acid) methanol, and injected on the HP1100/TSQ 7000 LC/MS system and gradient-eluted from the BDS Hypersil C8, 150 × 3.2 mm, 3-μm particle size column, with a mobile phase system consisting of 1.0 mM methanolic ammonium formate/2 mM aqueous ammonium formate. Peaks corresponding to the target analytes and IS were collected and processed using the Xcalibur software. Quantitative analyses of endogenous sphingolipids (SPLs) were based on calibration curves generated by spiking an artificial matrix with known amounts of the synthetic target analyte standards and an equal amount of the IS. The target analyte/IS peak area ratios were compared to the calibration curves using a linear regression model. Levels of a particular SPL were normalized to inorganic phosphate (Pi) released from total phospholipids, and expressed as SPL/Pi (pmol/μmol). Pi was released from phospholipids by acid hydrolysis and quantified as described [52, 53].

### Protein concentration determination

Protein concentrations were determined with BSA as a standard using a BCA protein determination kit (Pierce) according to the manufacturer's instructions.

### Ceramidase activity assay

Total membranes were isolated from cells and were assayed for ceramidase activity assays with different ceramides or ceramide analogs as substrates as described in our previous study [54]. Briefly, acid ceramidase activity encoded by ASAH1 was assayed at pH 4.5 using D-e-C_12_-ceramide as a substrate. Neutral ceramidase activity encoded by ASAH2 was assayed at pH 7.4 using D-*e*-C_12_-NBD as a substrate in the absence of Ca^2+^. Alkaline ceramidase activity encoded by ACER1, ACER2, or ACER3 was assayed at pH 8.5 in the presence of 5 mM Ca^2+^ using D-*e*-C_12_-NBD-ceramide, D-*e*-C_16_-ceramide, or D-*ribo*-C_12_-NBD-ceramide, respectively, as a substrate. SPH released from regular ceramides was determined by LC-MS/MS and NBD-fatty acid released from D-*e*-C_12_-NBD-ceramide or D-*ribo*-C_12_-NBD-ceramide was determined by thin liquid chromatography (TLC).

### Western blot analysis

Proteins were separated on SDS-polyacrylamide gels and transferred onto nitrocellulose membranes, which were then incubated with a primary antibody followed by a secondary antibody-conjugated with horseradish peroxidase (HRP). Protein bands were detected with VisGlo™ Prime HRP Chemiluminescent Substrate Kits (Amresco LLC, Solon, OH) according to the manufacturer's instructions. Protein band density was determined by densitometry performed on the imaging system Typhoon FLA 7000 (GE Healthcare Life Sciences; Pittsburgh, PA) according to the manufacturer's instructions.

### MTT assay

The number of viable cells was determined using an *in vitro* toxicology assay kit (MTT-based; Sigma, St. Louis, MO, USA) according to the manufacturer's instructions.

### LDH release assay

Plasma membrane permeability was assessed by the release of lactate dehydrogenase (LDH) from cells using Cytotoxicity Detection Kit PLUS (Roche Life Science) according to the manufacturer's instructions.

### Flow cytometry

Flow cytometry was performed as described in our previous study [30]. Briefly, cells were harvested by treatment with a trypsin-EDTA solution and washed with PBS. The harvested cells were fixed with 70% ethanol cooled at −20°C, treated with DNase-free RNase, and stained with propidium iodide (PI) before being analyzed by fluorescence activated cell sorting (FACS) on a FACStarplus flow cytometer (BD Biosciences) according to the manufacturer's instructions.

### Propidium iodide and alexa aluor 488-annexin V (PI/AA) co-staining

To detect apoptotic or necrotic cell death, cells were harvested by treatment with a trypsin-EDTA solution, washed with PBS, and stained with annexin V conjugated with Alexa Fluor 488 (AA) followed by propidium iodide (PI) using Alexa Fluor^®^ 488 Annexin V/Dead Cell Apoptosis Kits (ThermoFisher Scientific, Waltham, MA USA) according to the manufacturer's instructions. The stained cells were examined under a fluorescent microscope to enumerate cells (200 in total per sample) that were stained positively by AA (green fluorescence) and/or PI (red fluorescence).

### Caspase 3 activity assay

Caspase 3 activity was determined using DEVD-conjugated with 7-amino-4-trifluoromethyl coumarin (AFC) (R & D Systems, Inc., Minneapolis, MN) according to manufacturer's instructions.

### ROS assay

Intracellular ROS levels were measured with cell-permeable fluorogenic probe 2′, 7′-dichlorodihydrofluorescin diacetate (DCFH-DA) using OxiSelect™ Intracellular ROS Assay Kits (Green Fluorescence) (Cell Biolab Inc., San Diego, CA, USA) according to the manufacturer's instructions. Briefly, after being washed with PBS twice, cells were incubated with DCFH-DA-containing medium at 37°C for 30 minutes. The cells were then washed with PBS 3 times and lysed in a cell lysis buffer provided by the manufacturer before cell lysates were measured for fluorescence with a fluorometric plate reader at 480 nm/530 nm.

### Statistic analysis

Data are presented as the mean ± SD and were compared by one-way ANOVA (ANanlysis Of VAriance) with post hoc Tukey HSD (Honestly Significant Difference) using Graphpad Prism (La Jolla, CA). *p* values *p* < 0.05 were considered significant and marked with an asterisk (*) or #.

## SUPPLEMENTARY MATERIALS FIGURES



## References

[1] Su TT (2006). Cellular responses to DNA damage: one signal, multiple choices. Annual review of genetics.

[2] Nyberg KA, Michelson RJ, Putnam CW, Weinert TA (2002). Toward maintaining the genome: DNA damage and replication checkpoints. Annual review of genetics.

[3] Khanna A (2015). DNA Damage in Cancer Therapeutics: A Boon or a Curse?. Cancer Res.

[4] Uchida Y, Nardo AD, Collins V, Elias PM, Holleran WM (2003). *De novo* ceramide synthesis participates in the ultraviolet B irradiation-induced apoptosis in undifferentiated cultured human keratinocytes. J Invest Dermatol.

[5] Rotolo JA, Zhang J, Donepudi M, Lee H, Fuks Z, Kolesnick R (2005). Caspase-dependent and -independent activation of acid sphingomyelinase signaling. J Biol Chem.

[6] Mullen TD, Jenkins RW, Clarke CJ, Bielawski J, Hannun YA, Obeid LM (2011). Ceramide synthase-dependent ceramide generation and programmed cell death: involvement of salvage pathway in regulating postmitochondrial events. J Biol Chem.

[7] Hara S, Nakashima S, Kiyono T, Sawada M, Yoshimura S, Iwama T, Banno Y, Shinoda J, Sakai N (2004). p53-Independent ceramide formation in human glioma cells during gamma-radiation-induced apoptosis. Cell Death Differ.

[8] Chmura SJ, Nodzenski E, Beckett MA, Kufe DW, Quintans J, Weichselbaum RR (1997). Loss of ceramide production confers resistance to radiation-induced apoptosis. Cancer Res.

[9] Haimovitz-Friedman A, Kan CC, Ehleiter D, Persaud RS, McLoughlin M, Fuks Z, Kolesnick RN (1994). Ionizing radiation acts on cellular membranes to generate ceramide and initiate apoptosis. J Exp Med.

[10] Rotolo JA, Mesicek J, Maj J, Truman JP, Haimovitz-Friedman A, Kolesnick R, Fuks Z (2010). Regulation of ceramide synthase-mediated crypt epithelium apoptosis by DNA damage repair enzymes. Cancer Res.

[11] Senkal CE, Ponnusamy S, Rossi MJ, Bialewski J, Sinha D, Jiang JC, Jazwinski SM, Hannun YA, Ogretmen B (2007). Role of human longevity assurance gene 1 and C18-ceramide in chemotherapy-induced cell death in human head and neck squamous cell carcinomas. Mol Cancer Ther.

[12] Perry DK, Carton J, Shah AK, Meredith F, Uhlinger DJ, Hannun YA (2000). Serine palmitoyltransferase regulates *de novo* ceramide generation during etoposide-induced apoptosis. J Biol Chem.

[13] Mesicek J, Lee H, Feldman T, Jiang X, Skobeleva A, Berdyshev EV, Haimovitz-Friedman A, Fuks Z, Kolesnick R (2010). Ceramide synthases 2, 5, and 6 confer distinct roles in radiation-induced apoptosis in HeLa cells. Cell Signal.

[14] Dai Q, Liu J, Chen J, Durrant D, McIntyre TM, Lee RM (2004). Mitochondrial ceramide increases in UV-irradiated HeLa cells and is mainly derived from hydrolysis of sphingomyelin. Oncogene.

[15] Cuvillier O, Nava VE, Murthy SK, Edsall LC, Levade T, Milstien S, Spiegel S (2001). Sphingosine generation, cytochrome c release, and activation of caspase-7 in doxorubicin-induced apoptosis of MCF7 breast adenocarcinoma cells. Cell Death Differ.

[16] Gude DR, Alvarez SE, Paugh SW, Mitra P, Yu J, Griffiths R, Barbour SE, Milstien S, Spiegel S (2008). Apoptosis induces expression of sphingosine kinase 1 to release sphingosine-1-phosphate as a “come-and-get-me” signal. Faseb J.

[17] Weigert A, Johann AM, von Knethen A, Schmidt H, Geisslinger G, Brune B (2006). Apoptotic cells promote macrophage survival by releasing the antiapoptotic mediator sphingosine-1-phosphate. Blood.

[18] Greenspon J, Li R, Xiao L, Rao JN, Marasa BS, Strauch ED, Wang JY, Turner DJ (2009). Sphingosine-1-phosphate protects intestinal epithelial cells from apoptosis through the Akt signaling pathway. Digestive diseases and sciences.

[19] Schnitzer SE, Weigert A, Zhou J, Brune B (2009). Hypoxia enhances sphingosine kinase 2 activity and provokes sphingosine-1-phosphate-mediated chemoresistance in A549 lung cancer cells. Mol Cancer Res.

[20] Shin JH, Choi GS, Kang WH, Myung KB (2007). Sphingosine 1-phosphate triggers apoptotic signal for B16 melanoma cells via ERK and caspase activation. J Korean Med Sci.

[21] Chipuk JE, McStay GP, Bharti A, Kuwana T, Clarke CJ, Siskind LJ, Obeid LM, Green DR (2012). Sphingolipid metabolism cooperates with BAK and BAX to promote the mitochondrial pathway of apoptosis. Cell.

[22] Marchesini N, Hannun YA (2004). Acid and neutral sphingomyelinases: roles and mechanisms of regulation. Biochemistry and cell biology = Biochimie et biologie cellulaire.

[23] Stiban J, Tidhar R, Futerman AH (2010). Ceramide synthases: roles in cell physiology and signaling. Adv Exp Med Biol.

[24] Mao C, Obeid LM (2008). Ceramidases: regulators of cellular responses mediated by ceramide, sphingosine, and sphingosine-1-phosphate. Biochim Biophys Acta.

[25] Liu H, Chakravarty D, Maceyka M, Milstien S, Spiegel S (2002). Sphingosine kinases: a novel family of lipid kinases. Prog Nucleic Acid Res Mol Biol.

[26] Morales A, Paris R, Villanueva A, Llacuna L, Garcia-Ruiz C, Fernandez-Checa JC (2007). Pharmacological inhibition or small interfering RNA targeting acid ceramidase sensitizes hepatoma cells to chemotherapy and reduces tumor growth *in vivo*. Oncogene.

[27] Cheng JC, Bai A, Beckham TH, Marrison ST, Yount CL, Young K, Lu P, Bartlett AM, Wu BX, Keane BJ, Armeson KE, Marshall DT, Keane TE (2013). Radiation-induced acid ceramidase confers prostate cancer resistance and tumor relapse. J Clin Invest.

[28] Wu BX, Zeidan YH, Hannun YA (2009). Downregulation of neutral ceramidase by gemcitabine: Implications for cell cycle regulation. Biochim Biophys Acta.

[29] Uchida Y, Houben E, Park K, Douangpanya S, Lee YM, Wu BX, Hannun YA, Radin NS, Elias PM, Holleran WM (2010). Hydrolytic pathway protects against ceramide-induced apoptosis in keratinocytes exposed to UVB. J Invest Dermatol.

[30] Xu R, Jin J, Hu W, Sun W, Bielawski J, Szulc Z, Taha T, Obeid LM, Mao C (2006). Golgi alkaline ceramidase regulates cell proliferation and survival by controlling levels of sphingosine and S1P. Faseb J.

[31] Clarke CJ, Mediwala K, Jenkins RW, Sutton CA, Tholanikunnel BG, Hannun YA (2011). Neutral sphingomyelinase-2 mediates growth arrest by retinoic acid through modulation of ribosomal S6 kinase. J Biol Chem.

[32] Baumgartner A, Schmid TE, Cemeli E, Anderson D (2004). Parallel evaluation of doxorubicin-induced genetic damage in human lymphocytes and sperm using the comet assay and spectral karyotyping. Mutagenesis.

[33] Schnute ME, McReynolds MD, Kasten T, Yates M, Jerome G, Rains JW, Hall T, Chrencik J, Kraus M, Cronin CN, Saabye M, Highkin MK, Broadus R (2012). Modulation of cellular S1P levels with a novel, potent and specific inhibitor of sphingosine kinase-1. Biochem J.

[34] Hu W, Xu R, Zhang G, Jin J, Szulc ZM, Bielawski J, Hannun YA, Obeid LM, Mao C (2005). Golgi Fragmentation Is Associated with Ceramide-induced Cellular Effects. Mol Biol Cell.

[35] Kim HL, Han M, Im DS (2008). Differential signaling of sphingosine derivatives in U937 human monocytes depends on the degree of N-methylation. Prostaglandins and other lipid mediators.

[36] Abrahan C, Miranda G, Agnolazza D, Politi L, Rotstein N (2010). Synthesis of Sphingosine Is Required for Oxidative Stress-Induced Apoptosis of Photoreceptors. Invest Ophthalmol Vis Sci.

[37] Park MT, Kim MJ, Kang YH, Choi SY, Lee JH, Choi JA, Kang CM, Cho CK, Kang S, Bae S, Lee YS, Chung HY, Lee SJ (2005). Phytosphingosine in combination with ionizing radiation enhances apoptotic cell death in radiation-resistant cancer cells through ROS-dependent and -independent AIF release. Blood.

[38] Sun W, Jin J, Xu R, Hu W, Szulc ZM, Bielawski J, Obeid LM, Mao C (2010). Substrate specificity, membrane topology, and activity regulation of human alkaline ceramidase 2 (ACER2). J Biol Chem.

[39] Mao Z, Sun W, Xu R, Novgorodov S, Szulc ZM, Bielawski J, Obeid LM, Mao C (2010). Alkaline ceramidase 2 (ACER2) and its product dihydrosphingosine mediate the cytotoxicity of N-(4-hydroxyphenyl)retinamide in tumor cells. J Biol Chem.

[40] Mahdy AE, Cheng JC, Li J, Elojeimy S, Meacham WD, Turner LS, Bai A, Gault CR, McPherson AS, Garcia N, Beckham TH, Saad A, Bielawska A (2009). Acid ceramidase upregulation in prostate cancer cells confers resistance to radiation: AC inhibition, a potential radiosensitizer. Mol Ther.

[41] Haynes TA, Filippov V, Filippova M, Yang J, Zhang K, Duerksen-Hughes PJ (2012). DNA damage induces down-regulation of UDP-glucose ceramide glucosyltransferase, increases ceramide levels and triggers apoptosis in p53-deficient cancer cells. Biochim Biophys Acta.

[42] Lepine S, Lakatos B, Courageot MP, Le Stunff H, Sulpice JC, Giraud F (2004). Sphingosine contributes to glucocorticoid-induced apoptosis of thymocytes independently of the mitochondrial pathway. J Immunol.

[43] Suzuki E, Handa K, Toledo MS, Hakomori S (2004). Sphingosine-dependent apoptosis: a unified concept based on multiple mechanisms operating in concert. Proc Natl Acad Sci U S A.

[44] Nemoto S, Nakamura M, Osawa Y, Kono S, Itoh Y, Okano Y, Murate T, Hara A, Ueda H, Nozawa Y, Banno Y (2009). Sphingosine kinase isoforms regulate oxaliplatin sensitivity of human colon cancer cells through ceramide accumulation and Akt activation. J Biol Chem.

[45] Hazar-Rethinam M, de Long LM, Gannon OM, Topkas E, Boros S, Vargas AC, Dzienis M, Mukhopadhyay P, Simpson F, Endo-Munoz L, Saunders NA (2015). A novel E2F/sphingosine kinase 1 axis regulates anthracycline response in squamous cell carcinoma. Clin Cancer Res.

[46] Huwiler A, Kotelevets N, Xin C, Pastukhov O, Pfeilschifter J, Zangemeister-Wittke U (2011). Loss of sphingosine kinase-1 in carcinoma cells increases formation of reactive oxygen species and sensitivity to doxorubicin-induced DNA damage. British journal of pharmacology.

[47] Houben E, Holleran WM, Yaginuma T, Mao C, Obeid LM, Rogiers V, Takagi Y, Elias PM, Uchida Y (2006). Differentiation-associated expression of ceramidase isoforms in cultured keratinocytes and epidermis. J Lipid Res.

[48] Toman RE, Movsesyan V, Murthy SK, Milstien S, Spiegel S, Faden AI (2002). Ceramide-induced cell death in primary neuronal cultures: upregulation of ceramide levels during neuronal apoptosis. Journal of neuroscience research.

[49] Zigdon H, Kogot-Levin A, Park JW, Goldschmidt R, Kelly S, Merrill AH, Scherz A, Pewzner-Jung Y, Saada A, Futerman AH (2013). Ablation of ceramide synthase 2 causes chronic oxidative stress due to disruption of the mitochondrial respiratory chain. J Biol Chem.

[50] Sun W, Hu W, Xu R, Jin J, Szulc ZM, Zhang G, Galadari SH, Obeid LM, Mao C (2009). Alkaline ceramidase 2 regulates beta1 integrin maturation and cell adhesion. Faseb J.

[51] Muller P, Janovjak H, Miserez A, Dobbie Z (2002). Processing of Gene Expression Data Generated by Quantitative Real Time RT-PCR. BioTechniques.

[52] Murphy J, R JP (1962). A Modified Single Solution Method for the Dermination of Phosphate in Natural Waters. Analtyic Chimica Acta.

[53] Cogan EB, Birrell GB, Griffith OH (1999). A robotics-based automated assay for inorganic and organic phosphates. Analytical biochemistry.

[54] Xu R, Sun W, Jin J, Obeid LM, Mao C (2010). Role of alkaline ceramidases in the generation of sphingosine and its phosphate in erythrocytes. FASEB J.

