# Supplementary material for: Alkaline ceramidase 2 and its bioactive product sphingosine are novel regulators of the DNA damage response
Source: Oncotarget. 2016 Mar 1;7(14):18440–57. doi: 10.18632/oncotarget.7825 (PMC4951300; doi:10.18632/oncotarget.7825)
Supplement: Supplementary file 1 [file oncotarget-07-18440-s001.pdf]

Alkaline ceramidase 2 and its bioactive product sphingosine are novel regulators of the DNA damage response

Supplementary Materials

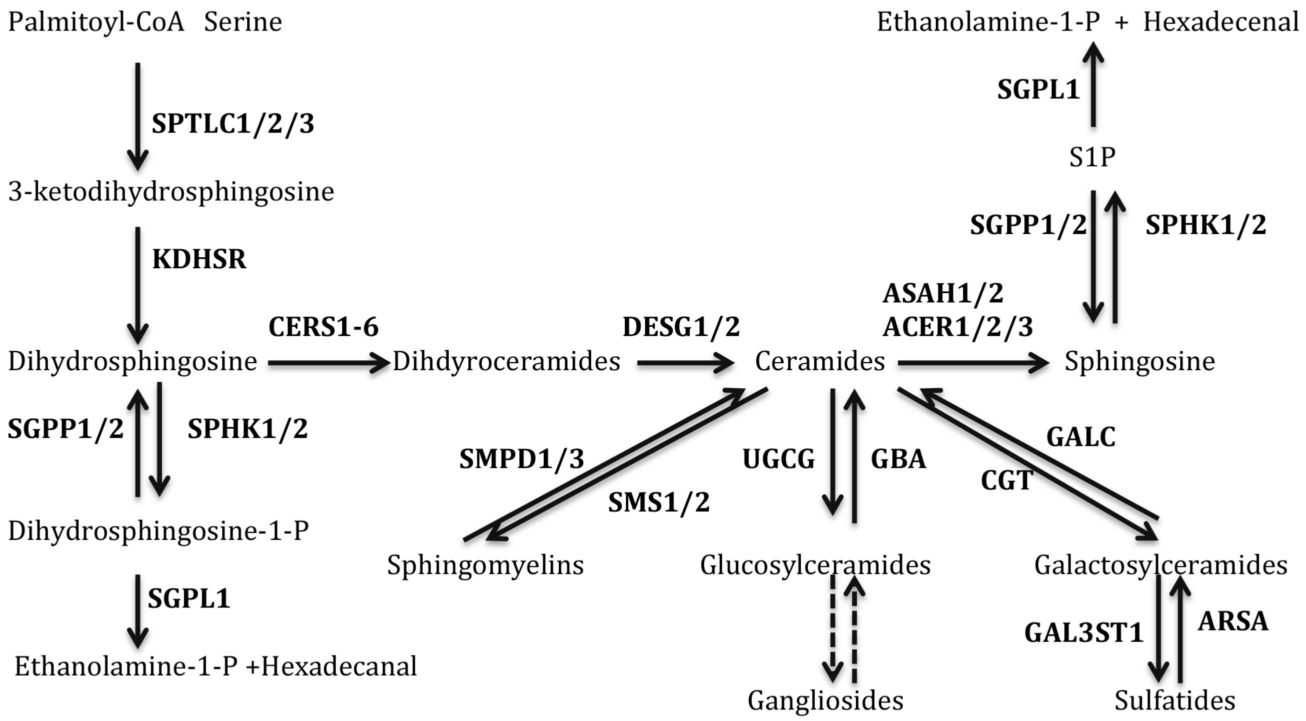

Supplementary Figure S1: Sphingolipid metabolism. SPTLC1/2/3, serine palmitoyltransferase subunit 1, 2, and 3; KDHSR, 3-keto-dihydrosphingosine reductase; CERS, (dihydro)ceramide synthase; DESG, dihydroceramide desaturase; ASAH1, acid ceramidase; ASAH2, neutral ceramidase; ACER, alkaline ceramidase; SMS, sphingomyelin synthase; UGCG, glucosylceramide synthase; CGT, galactosylceramide synthase; GAL3ST1, Galactose-3-O-sulfotransferase 1, SMPD1, acid sphingomyelinase; SMPD3, neutral sphingomyelinase 2; GBA, beta-glucocerebrosidase; ARSA, arylsulfatase A; GALC, galactocerebroside beta-Galactosidase; SPHK, sphingosine kinase; SGPP, S1P phosphohydrolase; and SGPL, S1P lyase.

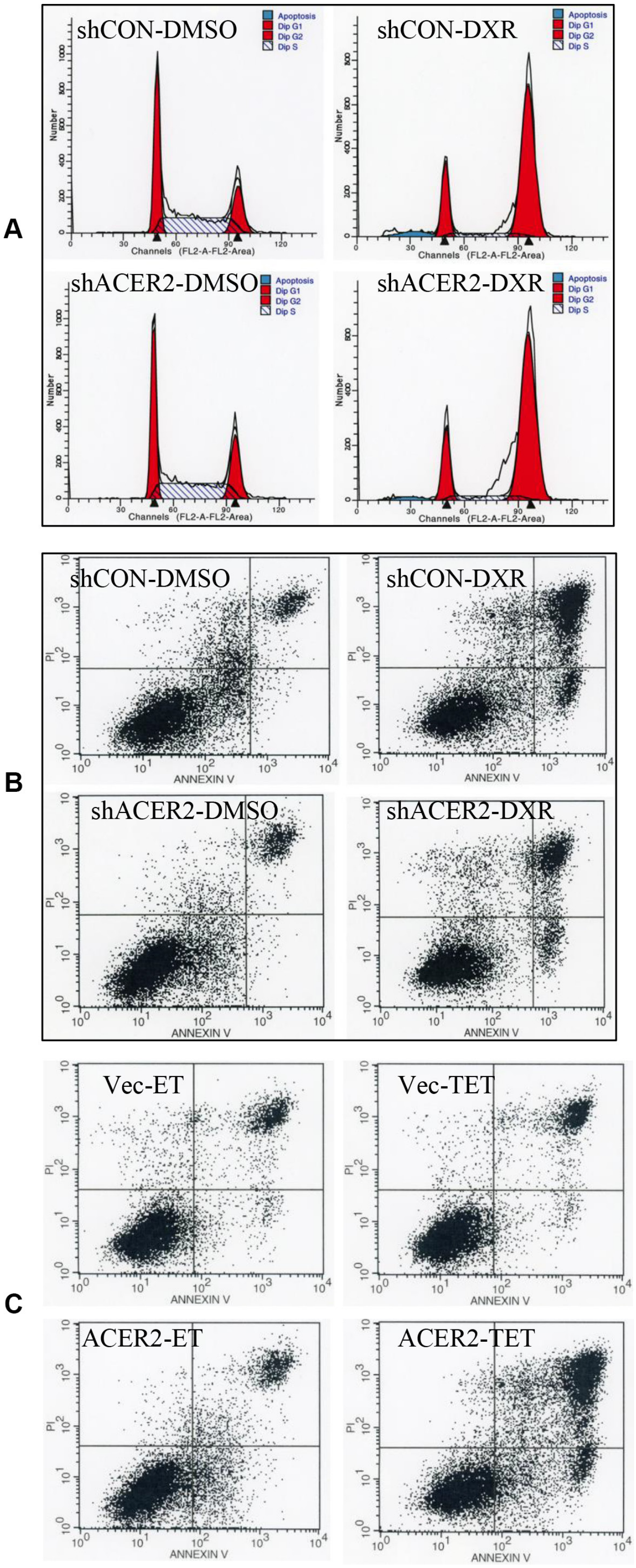

Supplementary Figure S2: ACER2 knockdown inhibits programmed cell death but not cell cycle arrest of HCT116 cells in response to DXR whereas ACER2 overexpression induces programmed cell death. HCT116 cells stably expressing shCON or shACER2 were treated with DXR at 400 nM (a) before cell cycle progression was determined by flow cytometry (**A**). HCT116 cells stably expressing shCON or shACER2 were treated with DXR at 1,000 nM before cell death was assessed by PI/AA co-staining followed by flow cytometry (**B**). VEC-TET-ON or haCER2-TET-ON cells were grown to a 25% confluence before they were treated with TET (20 ng/ml) or ET. At 72 h post treatment with ET or TET, cell death was assessed by PI/AA co-staining followed by flow cytometry.

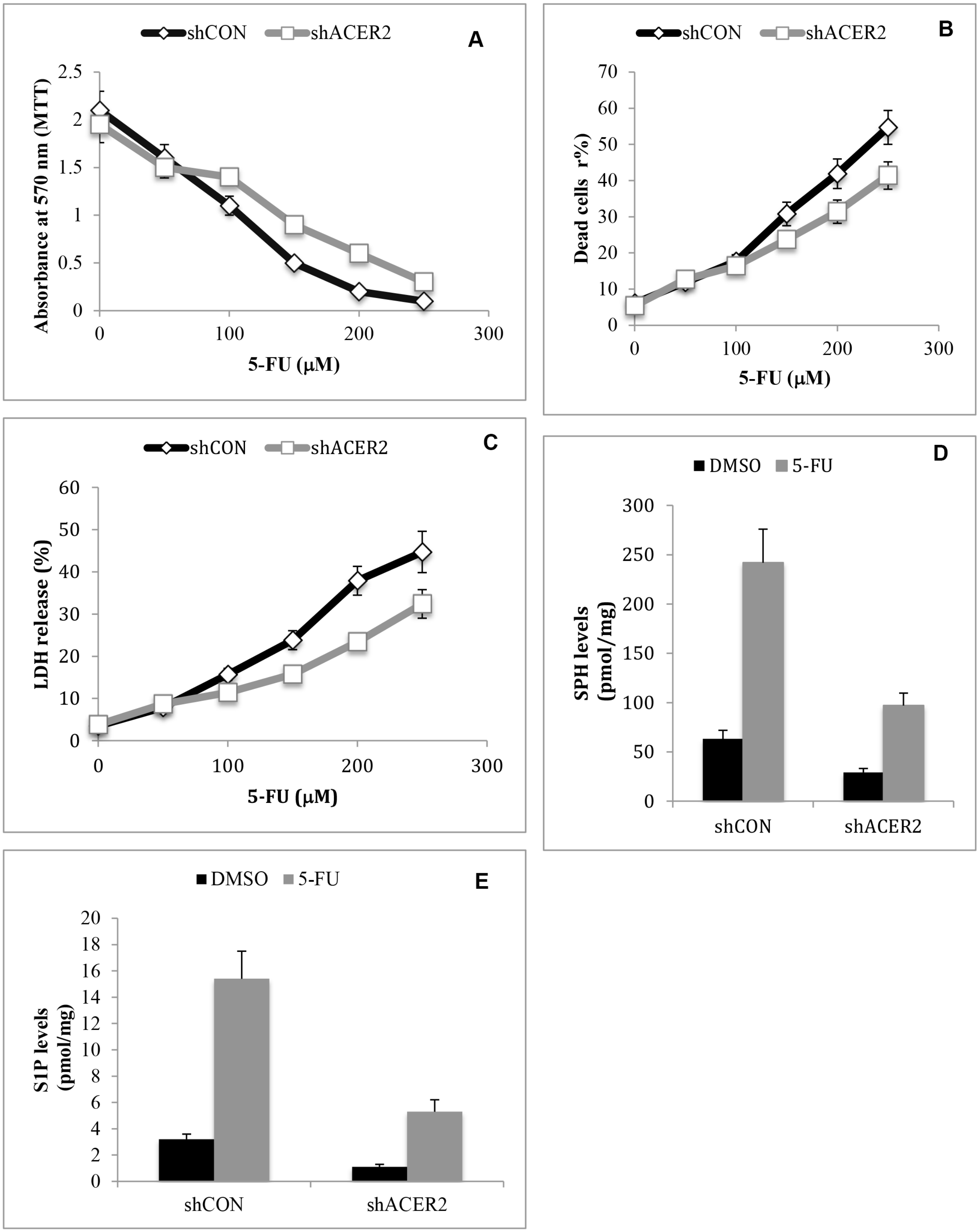

Supplementary Figure S3: ACER2 knockdown inhibits SPH generation and programmed cell death in HCT116 cells in response to treatment with 5-FU. HCT116 cells stably expressing shCON or shACER2 were treated with 5-FU at 300 nM or DMSO for 48 h before being subjected to SPH measurement by LC-MS/MS (**A**), MTT assays (**B**), PI/AA co-staining (**C**) or LDH assays (**D**). Data represent mean values ± SD of 3 independent experiments.

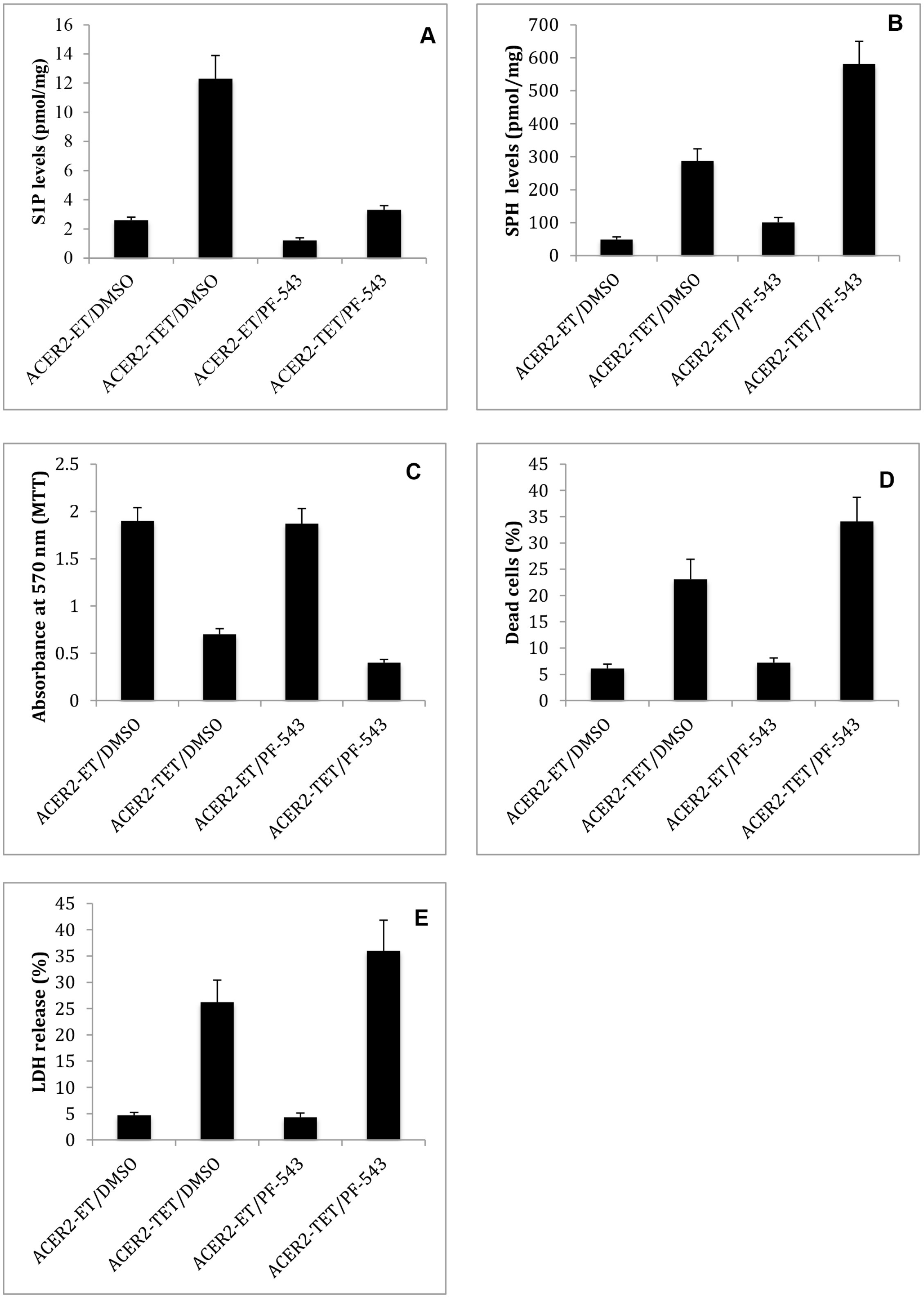

Supplementary Figure S4: Inhibiting SPHK1 with PF543 enhances PCD in response to ACER2 overexpression. haCER2-TET-ON cells at a 25% confluence were treated with PF543 (100 nM) or DMSO for 2 h before they were treated with ET or TET (20 ng/ml) for 72 h. Post ET or TET treatment, cells were subjected to LC-MS/MS analyses for the levels of S1P (**A**) and SPH (**B**), MTT assays (**C**), PI/AA co-staining assays (**D**), or LDH release assays (**E**). Data represent mean values ± SD of 3 independent experiments.
